# Oral, intravenous, or sequential alpha-lipoic acid for diabetic peripheral neuropathy? A Bayesian network meta-analysis of randomized controlled trials

**DOI:** 10.3389/fneur.2026.1904967

**Published:** 2026-08-11

**Authors:** Jinshui Shen, YuJing Zheng, MiaoMiao Yu, Xu Wang

**Affiliations:** The First School of Clinical Medicine, Nanjing University of Chinese Medicine, Nanjing, China

**Keywords:** administration routes, alpha-lipoic acid, diabetic peripheral neuropathy, network meta-analysis, randomized controlled trials, sequential therapy

## Abstract

**Objective:**

Alpha-lipoic acid (ALA) is widely utilized for diabetic peripheral neuropathy (DPN); however, optimal administration routes (oral, intravenous [IV], or sequential) remain debated. This study evaluates and ranks their efficacy and safety through a Bayesian network meta-analysis (NMA) equipped with rigorous bias-exclusion frameworks.

**Methods:**

The PubMed, Embase, Web of Science, Cochrane Library, and Scopus databases were systematically searched (up to 2025) to identify randomized controlled trials (RCTs) comparing oral, intravenous, and sequential ALA therapies, as well as placebo, encompassing varying dosages (600–1,800 mg/d) and treatment durations (3 to 208 weeks), for DPN. Core outcome measures included the Total Symptom Score (TSS), Neuropathy Impairment Score (NIS), Neuropathy Impairment Score in the Lower Limbs (NIS-LL), and Global Satisfaction (GS). The NMA was conducted using a Bayesian framework in R software. Interventions were ranked by calculating the surface under the cumulative ranking curve (SUCRA), and a dual-outcome plot was constructed to evaluate the benefit–risk ratio. Evidence certainty was evaluated via CINeMA, and sensitivity analyses were executed by excluding high-bias studies.

**Results:**

Nine high-quality RCTs were included. Initially, sequential ALA therapy demonstrated overwhelming superiority in improving TSS and NIS. However, CINeMA evaluations revealed severe within-study bias, and sensitivity analyses exposed this initial superiority as an artifact. In the unbiased network, IV ALA emerged as the absolute optimal intervention for rapidly alleviating subjective symptoms (TSS) and overall objective signs (NIS), achieving a “dual-optimal” efficacy-safety profile that completely bypasses gastrointestinal risks. Confronting the most refractory distal impairment (NIS-LL), oral ALA stood alone as the sole surviving intervention supported by robust, completely homogeneous evidence (I^2^ = 0%, SUCRA: 97.4%) for structural repair. Both standalone IV and oral routes significantly enhanced global patient satisfaction.

**Conclusion:**

The optimal ALA administration is stage-dependent. IV ALA delivers unmatched acute neurovascular rescue, while oral ALA serves as the indispensable cornerstone for long-term distal structural repair. Importantly, rather than negating sequential therapy, these distinct phase-specific benefits fundamentally validate its core “induction-maintenance” clinical rationale. While unbiased evidence for the integrated sequential regimen remains sparse—necessitating future large-scale, double-blinded RCTs—the sequential framework itself is robustly justified by the verified strengths of its constituent phases.

**Systematic review registration:**

CRD420261411001.

## 1 Introduction

DPN is one of the most common and insidious chronic complications of diabetes mellitus, primarily manifesting as symmetric, length-dependent distal sensory nerve damage. Patients frequently present with numbness, burning pain, prickling sensations, paresthesia, and hypoesthesia in the feet and lower extremities, with lesions progressively advancing from the distal feet to proximal regions (1). Because early-stage DPN is frequently underdiagnosed, the subsequent loss of protective sensation significantly exacerbates the risk of foot ulcers, infections, non-traumatic amputations, and mortality. This progression profoundly impairs long-term quality of life and imposes a severe medical and socioeconomic burden (2). Epidemiological studies indicate that the prevalence of DPN among patients with diabetes ranges from 20 to 50% (2). Furthermore, up to 50% of patients with type 1 or type 2 diabetes, alongside 30% of individuals with prediabetes, will eventually develop DPN (2, 3).

The pathogenesis of DPN is multifaceted, involving glucolipid metabolic disorders, insulin resistance, oxidative stress, chronic inflammation, mitochondrial dysfunction, and neural microvascular hypoperfusion. Among these factors, oxidative stress is recognized as a critical nexus linking metabolic abnormalities to nerve injury. Excessive reactive oxygen species (ROS) impair vascular endothelial function, reduce neural microcirculatory perfusion, and promote nerve fiber demyelination and axonal degeneration (4, 5). Simply controlling blood glucose levels yields limited efficacy in alleviating DPN symptoms or restoring nerve function; conventional treatments have primarily focused on symptom relief and rarely reverse the progression of nerve damage (6). ALA, a potent lipophilic free-radical scavenger, significantly improves ischemic neural microvascular perfusion and mitigates endothelial dysfunction by neutralizing tissue and blood-borne ROS and upregulating endothelial nitric oxide synthase expression (7). While ALA effectively ameliorates various neuropathological alterations in DPN, its optimal clinical application warrants systematic evaluation.

Over the past two decades, numerous large-scale, landmark RCTs, such as the ALADIN and SYDNEY series, have robustly demonstrated that ALA effectively improves core clinical outcomes in DPN patients. These include the subjective TSS and the objective NIS (8–10). However, current clinical practice exhibits significant heterogeneity in ALA intervention strategies, which primarily encompass ALA oral monotherapy (ALA Oral), ALA intravenous infusion (ALA IV), and ALA sequential therapy—defined as an initial intensive intravenous regimen followed by oral maintenance (ALA Sequential). These disparate administration routes profoundly influence the drug’s bioavailability, first-pass metabolism, and the maintenance of a sustained antioxidant microenvironment (11), thereby exerting a decisive impact on overall therapeutic efficacy and patient tolerability.

To date, there remains a global paucity of large-sample, high-quality RCTs conducting direct head-to-head comparisons among these four dosing strategies (oral, IV, sequential, and placebo). To bridge this crucial clinical research gap, this study—strictly adhering to the PRISMA-NMA reporting guidelines—employs a NMA methodology to integrate these four interventions into a connected Bayesian comparative framework for the first time. By synthesizing both direct and indirect evidence, we aim to systematically evaluate and comprehensively rank the differential efficacy of these varied administration routes in improving TSS, NIS, NIS-LL, and global satisfaction, thereby furnishing evidence-based, optimal dosing regimens for the future precision-stratified treatment of DPN.

## 2 Materials and methods

The design, execution, and reporting of this systematic review and NMA strictly adhered to the updated Preferred Reporting Items for Systematic Reviews and Meta-Analyses (PRISMA 2020) guidelines and the PRISMA-NMA extension. The study protocol was pre-registered in CRD420261411001.

### 2.1 Search strategy and eligibility criteria

To comprehensively capture relevant global clinical evidence, a systematic literature search was conducted across five core electronic databases—PubMed, Embase, Web of Science, Cochrane Library, and Scopus—from 1990 to 2025. The search strategy employed a combination of Medical Subject Headings (MeSH) and free-text terms; core terms included “alpha-lipoic acid,” “thioctic acid,” “diabetic peripheral neuropathy,” and “randomized controlled trials.” The detailed search strategies tailored for each database are provided in Supplementary Table S1.

Literature screening strictly conformed to the pre-specified PICOS framework: Population (P): Patients diagnosed with DPN, without restrictions on age, sex, or disease duration. Intervention (I): ALA Oral. Comparison (C): ALA IV, ALA Sequential (defined as an initial intravenous intensive regimen followed by oral maintenance), or placebo, establishing a comparative network of four nodal interventions. Outcomes (O): Studies were required to report at least one core outcome: continuous variables including the TSS, NIS, and Neuropathy Impairment Score in the Lower Limbs (NIS-LL), or dichotomous variables such as GS. Study Design (S): Only high-quality RCTs were eligible. Non-randomized studies, animal experiments, case reports, and literature lacking primary data were excluded.

Following screening based on these rigorous criteria, two investigators independently performed data extraction and quality assessment, ultimately including 9 high-quality RCTs (12–20).

### 2.2 Data extraction and risk of bias assessment

Two investigators independently extracted key baseline characteristics and outcome data from the eligible studies, including the first author, publication year, sample size, patient demographics, detailed intervention protocols (specifically capturing varying ALA dosages and treatment/follow-up durations), and core subjective and objective outcome measures. For multi-arm trials, data were partitioned and extracted in strict accordance with the network node definitions to preserve the validity of subsequent indirect comparisons.

Methodological quality was assessed using the Cochrane Risk of Bias tool 2.0 (RoB 2). Accounting for the varying susceptibility of subjective and objective outcomes to performance and detection bias, we performed a refined, outcome-specific assessment. The evaluated domains comprised random sequence generation, allocation concealment, deviations from intended interventions, missing outcome data, measurement of the outcome, and selection of the reported result. Any discrepancies were resolved through consensus or arbitration by a third senior investigator. Importantly, the results of this RoB 2.0 assessment directly informed our subsequent sensitivity analyses to test the robustness of the findings.

### 2.3 Statistical analysis

Data analysis integrated conventional pairwise meta-analyses with network meta-analyses, executed within the R Studio software environment.

First, for interventions with direct head-to-head evidence, pairwise meta-analyses were performed using the meta package. Mean differences (MDs) were calculated as effect sizes for continuous variables (TSS, NIS, and NIS-LL), while risk ratios (RRs) were used for dichotomous variables (GS). All estimates were accompanied by 95% confidence intervals (CIs) and pooled using a random-effects model. To thoroughly explore heterogeneity across the comparisons, we calculated both the Bayesian between-study variance (τ^2^) and frequentist pairwise statistics, including the I^2^ statistic and Cochran’s Q test.

For the NMA phase, the network model was fitted within a Bayesian framework using the Markov Chain Monte Carlo (MCMC) method via the gemtc and rjags packages. Network plots were generated to visualize the topological distribution of sample sizes and direct comparative evidence across nodes. For networks containing closed loops (TSS, NIS, and NIS-LL), the node-splitting method was applied to evaluate local inconsistency; for the sparse network lacking closed loops (GS), effect estimates were calculated using a consistency model.

To hierarchically rank the interventions, the surface under the cumulative ranking curve (SUCRA) was calculated, where higher SUCRA values indicate a greater probability that an intervention is optimal. To balance efficacy against safety, relative treatment effects and adverse event incidence rates were extracted to construct a dual-outcome quadrant plot.

Crucially, to rigorously validate the stability of our main findings and address the potential impact of within-study bias, a sensitivity analysis was executed. This involved excluding RCTs assessed as having a high risk of bias and re-running the entire NMA framework to verify if the SUCRA rankings and pooled estimates remained robust.

Finally, publication bias and network robustness were evaluated using comparison-adjusted funnel plots designed for NMA. To standardize the projection of effect sizes, a predefined treatment hierarchy was established (Placebo → ALA Oral → ALA Sequential → ALA IV). This sequence was strictly defined based on an escalation of treatment intensity, clinical invasiveness, and expected acute efficacy, under the rationale that small-study effects typically exaggerate the effect sizes of more invasive interventions. This funnel plot assessment was applied exclusively to continuous outcome networks with closed loops and sufficient nodal data (TSS, NIS, and NIS-LL); it was omitted for the sparse categorical outcome (GS) due to a lack of network loops and insufficient statistical power. Furthermore, visual inspections of funnel plot asymmetry were quantitatively verified using an Egger’s regression test analogue specifically adapted for NMA.

### 2.4 Certainty of evidence assessment

To comprehensively evaluate the robustness of the evidence underlying our NMA results and SUCRA rankings, the Confidence in Network Meta-Analysis (CINeMA) framework was employed. The certainty of evidence for each comparison across the core outcomes (TSS, NIS, NIS-LL, and GS) was graded as High, Moderate, Low, or Very Low. This grading was based on the evaluation of six specific domains: within-study bias, reporting bias, indirectness, imprecision, heterogeneity, and incoherence.

## 3 Results

### 3.1 Characteristics of included studies and risk of bias

#### 3.1.1 PRISMA

A comprehensive and systematic search was performed across five core electronic databases, initially identifying 1,314 relevant records: PubMed (*n* = 248), Embase (*n* = 390), Web of Science (*n* = 103), Cochrane Library (*n* = 176), and Scopus (*n* = 397). Prior to screening, 583 duplicate records were removed using Zotero reference management software, and 2 records were excluded due to retraction. Consequently, 729 records proceeded to the title and abstract screening phase.

Two independent investigators reviewed the titles and abstracts of these 729 records, excluding 635 that failed to meet the predefined inclusion criteria. Specific reasons for exclusion at this stage included: non-randomized controlled trials (non-RCTs, *n* = 294), ineligible study population (non-DPN, *n* = 126), ineligible intervention (non- ALA monotherapy therapy, *n* = 91), ineligible comparator (absence of either a placebo control or a head-to-head ALA-route comparison, *n* = 104), and lack of TSS outcome (*n* = 20).

Extensive efforts—encompassing database expansion, school library acquisitions, inter-library loans (ILL), and direct correspondence with authors—were undertaken to retrieve the full texts for the remaining 94 reports. However, 32 reports were not retrievable. Thus, 62 reports underwent a rigorous full-text eligibility assessment based on the PICOS framework.

A total of 53 reports were further excluded for the following specific reasons: non-RCT design (*n* = 16); ineligible population (Type 1 DPN or diabetic autonomic neuropathy, *n* = 5); ineligible intervention (e.g., ALA combined with pregabalin or acupuncture, *n* = 2); ineligible comparator (e.g., active controls such as amitriptyline, duloxetine, or pregabalin instead of placebo or ALA-route comparisons, *n* = 21); lack of relevant outcome measures (missing TSS or secondary metrics such as NIS, NISLL, satisfaction, or adverse events, *n* = 8); and insufficient data (one study provided only a narrative statement without extractable TSS data, *n* = 1).

**Figure 1:**
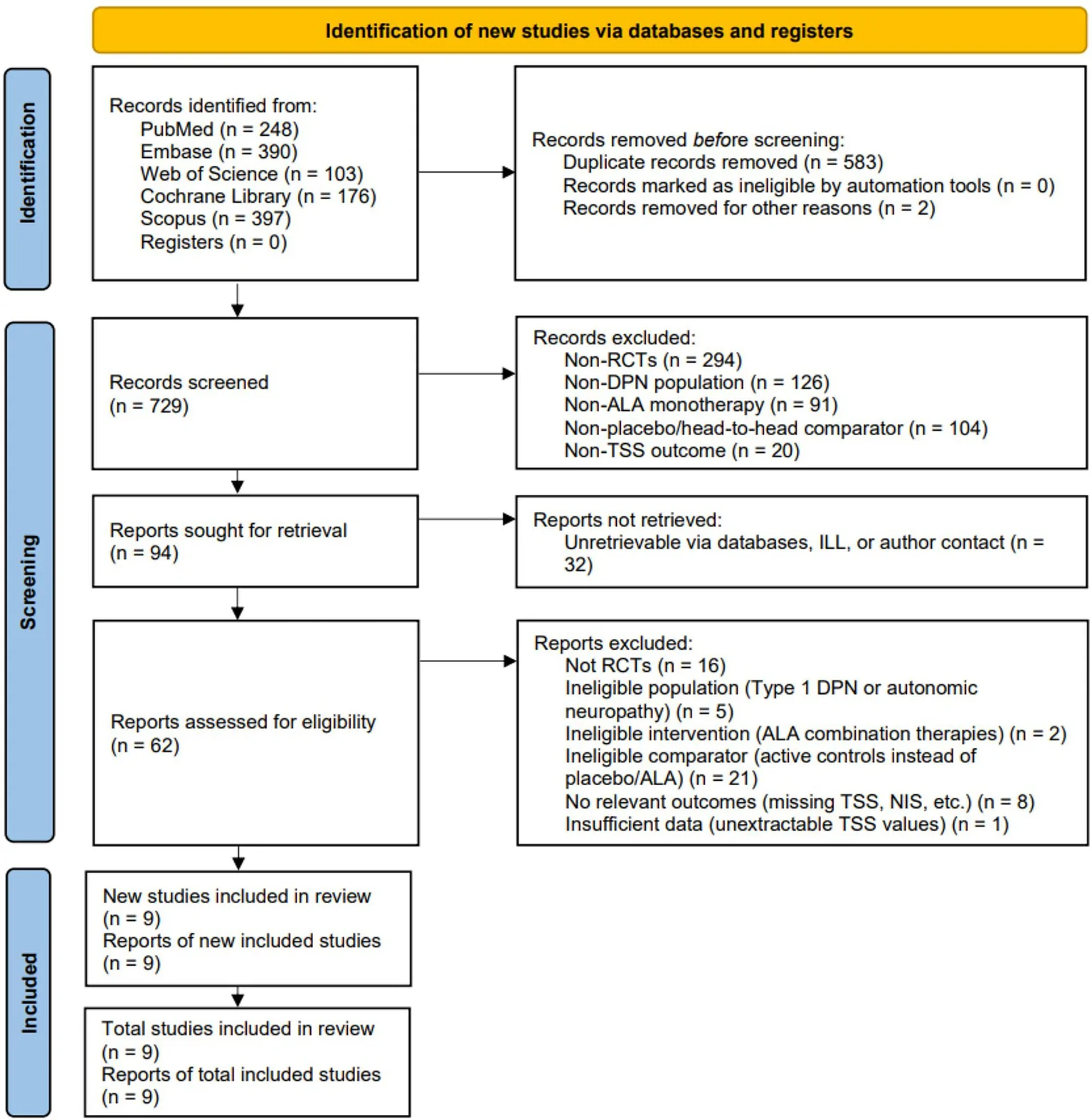
The PRISMA flow diagram.

Ultimately, 9 high-quality RCTs meeting all inclusion criteria were included in this systematic review and NMA. The complete identification and selection process is detailed in the PRISMA 2020 flow diagram (Figure 1).

#### 3.1.2 Baseline characteristics of the data

These 9 randomized controlled trials were conducted across multiple countries and regions, including Germany, Russia, Mexico, South Korea, Pakistan, and North America. They encompassed diverse study designs, ranging from single-center to multicenter, and double-blind to open-label trials, thereby ensuring the geographic and demographic representativeness of the sample. Overall, the baseline characteristics of all included patients were well-balanced. The mean age of patients across the intervention groups ranged from 46.1 to 62.1 years, and the mean duration of type 2 diabetes varied between 10.0 and 20.9 years, aligning precisely with the typical epidemiological profile of patients with diabetic peripheral neuropathy.

Regarding the intervention network, data from complex multi-arm trials were successfully extracted and partitioned into four distinct intervention nodes: oral ALA, intravenous ALA, sequential ALA therapy, and placebo.

In terms of dosage and administration routes, while 600 mg/day served as the most prevalent standard clinical dose—predominating the oral, IV, and sequential groups—the network also incorporated studies exploring various dosage gradients. For instance, Ziegler (18) evaluated oral dosages of 600, 1,200, and 1,800 mg/day; Ziegler (14) investigated IV dosages of 100, 600, and 1,200 mg/day; and Ruhnau (19) employed a high-dose oral regimen of 1,800 mg/day.

Regarding treatment duration, the intervention periods across the studies exhibited a broad span. These encompassed short-term regimens of 2 to 5 weeks designed to evaluate acute efficacy [e.g., (15, 16)], as well as extended follow-up periods ranging from 6 months to 4 years intended to assess long-term maintenance efficacy [e.g., (13, 17)].

Regarding outcome reporting, all included studies documented the subjective TSS, establishing a robust direct comparative evidence chain for this NMA. Furthermore, several large-scale trials [e.g., (13, 15, 18)] additionally reported the NIS, NIS-LL, and GS, thereby supplying multidimensional, multiple-outcome data for a comprehensive evaluation of the clinical benefits conferred by ALA.

Overall, the included studies demonstrated a high degree of consistency regarding core patient demographics and underlying disease characteristics. No significant baseline imbalances were observed, thus furnishing a reliable foundation of transitivity for subsequent indirect comparisons within the network meta-analysis. The baseline Characteristics of the Data is detailed in Table 1.

**Table 1:** The baseline characteristics of the data.

Study	Country	Study design	Therapy	*N*	Age	Course of T2DM	ALA regimen	Treatment duration	Outcomes
Garcia-Alcala, 2015 (12)	Mexico	Multicenter, enriched-enrollment, randomized-withdrawal, open-label RCT	ALA orally	16	57.5 ± 10.0	10.4 ± 7.8	600 mg/day orally for 16 weeks	16 weeks	TSS
Garcia-Alcala, 2015 (12)	Mexico	Multicenter, enriched-enrollment, randomized-withdrawal, open-label RCT	Placebo	17	59.0 ± 11.0	13.0 ± 5.0	Matching placebo	16 weeks	TSS
Ziegler, 1999 (13)	Germany	Multicenter, randomized, double-blind, placebo-controlled, sequential-treatment RCT	ALA sequentially	165	56.5 ± 7.1	11.5 ± 8.4	600 mg/day intravenously for 3 weeks followed by 600 mg/day orally for 5 months	6 months	TSS, NIS, NISLL
Ziegler, 1999 (13)	Germany	Multicenter, randomized, double-blind, placebo-controlled, sequential-treatment RCT	ALA intravenously	338	56.8 ± 6.7	11.6 ± 8.1	600 mg/day intravenously 3 weeks	3 weeks	TSS, NIS, NISLL
Ziegler, 1999 (13)	Germany	Multicenter, randomized, double-blind, placebo-controlled, sequential-treatment RCT	Placebo	165	57.3 ± 5.6	11.3 ± 7.7	Matching placebo	6 months	TSS, NIS, NISLL
Ziegler, 1995 (14)	Germany	Multicenter, randomized, double-blind, placebo-controlled, four-arm parallel-group RCT	ALA intravenously	194	58.5 ± 8.1	11.0 ± 7.1	100/600/1200 mg/day intravenously for 3 weeks	3 weeks	TSS, GS
Ziegler, 1995 (14)	Germany	Multicenter, randomized, double-blind, placebo-controlled, four-arm parallel-group RCT	Placebo	66	60.2 ± 7.7	12.3 ± 7.7	Matching placebo	3 weeks	TSS, GS
Ametov, 2003 (15)	Russia	Single-center, randomized, double-blind, parallel-group RCT	ALA intravenously	60	56.8 ± 9.7	15.1 ± 8.8	600 mg/day intravenously for 3 weeks	3 weeks	TSS, NIS, GS
Ametov, 2003 (15)	Russia	Single-center, randomized, double-blind, parallel-group RCT	Placebo	60	55.4 ± 8.7	14.0 ± 8.2	Matching placebo	3 weeks	TSS, NIS, GS
Lee, 2006 (16)	South Korea	Randomized, parallel-group controlled RCT	ALA intravenously	21	56.8 ± 10.7	10.8 ± 6.7	600 mg/day intravenously for 2 weeks	2 weeks	TSS
Lee, 2006 (16)	South Korea	Randomized, parallel-group controlled RCT	ALA sequentially	21	56.8 ± 10.7	10.8 ± 6.7	600 mg/day intravenously for 2 weeks followed by 600 mg/day orally for 10 weeks	12 weeks	TSS
Lee, 2006 (16)	South Korea	Randomized, parallel-group controlled RCT	ALA orally	24	58.9 ± 5.3	10.1 ± 6.3	600 mg/day orally for 12 weeks	12 weeks	TSS
Ziegler, 2011 (17)	The United States, Canada and Europe	Multicenter, randomized, double-blind, placebo-controlled, parallel-group RCT	ALA orally	230	53.3 ± 8.3	20.9 ± 9.2	600 mg/day orally for 4 years	4 years	TSS, NIS, NISLL
Ziegler, 2011 (17)	The United States, Canada and Europe	Multicenter, randomized, double-blind, placebo-controlled, parallel-group RCT	Placebo	224	53.9 ± 7.6	18.7 ± 7.7	Matching placebo	4 years	TSS, NIS, NISLL
Ziegler, 2006 (18)	Russia and Israel	Multicenter, randomized, double-blind, placebo-controlled, four-arm parallel-group RCT	ALA orally	138	58.0 ± 11.1	14.0 ± 9.7	600/1200/1800 mg orally for 5 weeks	5 weeks	TSS, NIS, NISLL, GS
Ziegler, 2006 (18)	Russia and Israel	Multicenter, randomized, double-blind, placebo-controlled, four-arm parallel-group RCT	Placebo	43	57.0 ± 11.0	14.0 ± 10.0	Matching placebo	5 weeks	TSS, NIS, NISLL, GS
Ruhnau, 1999 (19)	Germany	Multicenter, randomized, double-blind, placebo-controlled RCT	ALA orally	11	60.5 ± 6.9	10.6 ± 3.3	1800 mg/day orallay for 3 weeks	3 weeks	TSS
Ruhnau, 1999 (19)	Germany	Multicenter, randomized, double-blind, placebo-controlled RCT	Placebo	11	62.1 ± 4.5	12.4 ± 10.9	Matching placebo	3 weeks	TSS
Siddique, 2021 (20)	Pakistan	Single-center, randomized, open-label RCT	ALA orally	55	46.1 ± 11.9	11.2 ± 5.4	600 mg orally for 5 weeks	5 weeks	TSS
Siddique, 2021 (20)	Pakistan	Single-center, randomized, open-label RCT	Placebo	55	47.7 ± 10.7	10.0 ± 5.8	Matching placebo	5 weeks	TSS

#### 3.1.3 Risk of bias assessment

This study employed the Cochrane RoB 2 tool to evaluate the risk of bias in the 9 included RCTs. The methodological quality of the included studies varied: 3 studies (33.3%) were rated as having a low risk of bias, 2 studies (22.2%) were assessed as having some concerns, and 4 studies (44.4%) were deemed to have a high risk of bias.

The primary sources of bias were concentrated in specific areas. First, in the domains of “deviations from intended interventions” (Domain 2) and “measurement of the outcome” (Domain 4), approximately 33% (3 studies) were rated as high risk [e.g., (12, 16, 20)]. This was primarily because the TSS is a highly subjective symptomatology scale; the failure of certain studies to implement a rigorous double-blind design allowed patients and assessors to be aware of the intervention assignments, thereby introducing substantial performance and detection biases.

Second, concerning the “randomization process” (Domain 1), although all studies were described as randomized, nearly half were evaluated as having some concerns [e.g., (18, 19)] or a high risk of bias due to insufficient reporting on the specific methods used for random sequence generation or allocation concealment.

Finally, regarding “missing outcome data” (Domain 3), the overall performance was satisfactory, with approximately 78% of studies evaluated as low risk. However, two studies (13, 18) were rated as high risk due to elevated loss-to-follow-up rates and the failure to employ an appropriate intention-to-treat (ITT) analysis to handle missing values.

**Figure 2:**
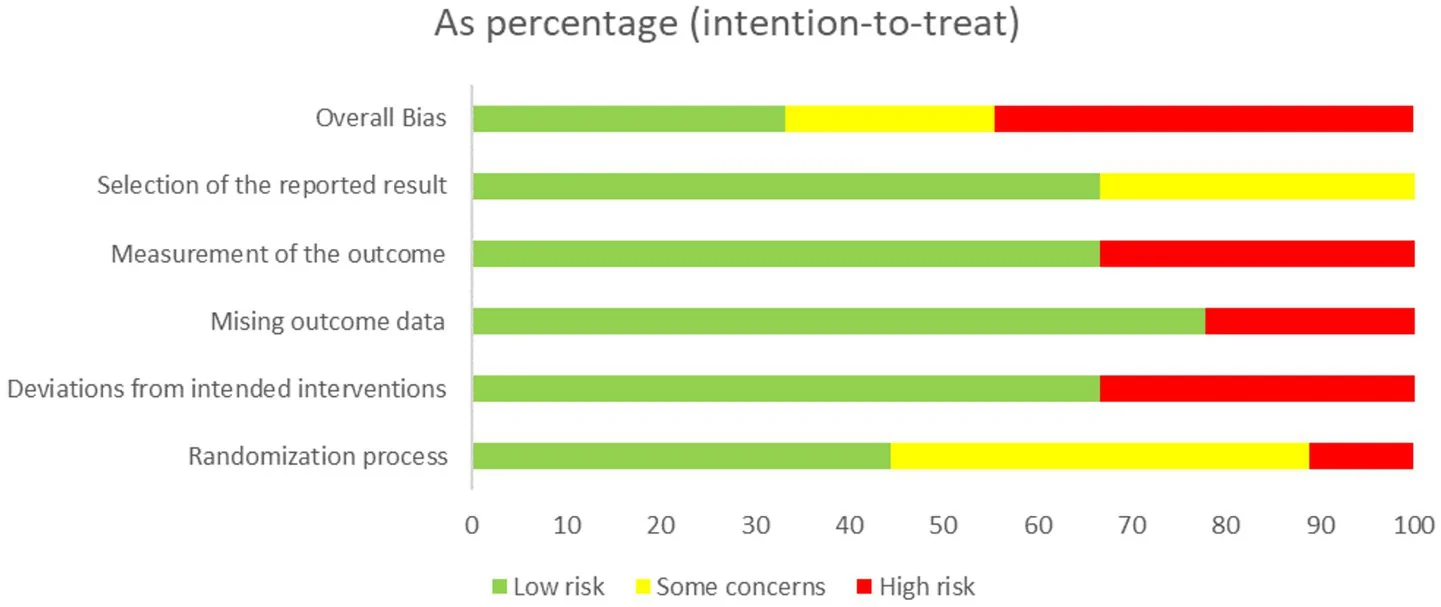
Risk of bias assessment.

Despite the methodological flaws present in certain trials, the remaining low-risk, large-scale studies provided a solid and robust foundation of direct evidence to support the indirect comparison network. The Risk of Bias Assessment is detailed in Figure 2.

### 3.2 Network evidence plots and pairwise meta-analysis

#### 3.2.1 TSS

For the core subjective outcome measure, the TSS, we constructed a closed-loop network comparison system comprising four intervention nodes: placebo, ALA oral， ALA IV, and ALA sequential therapy. Regarding network geometry, all interventions were interconnected, forming a complete evidence network that established the statistical foundation for conducting mixed direct and indirect comparisons. In terms of evidence distribution, significant asymmetry was observed in node sizes (representing total sample sizes) and edge thicknesses (representing the number of direct comparative studies).

Placebo served as the common control group, constituting the central hub of the network. Among the intervention groups, the oral ALA node was the largest and featured the thickest edge connecting it to the placebo, indicating that it possessed the largest sample size and the most abundant support from direct head-to-head comparative evidence. This was followed by the IV ALA group. Conversely, the sequential ALA therapy node was the smallest with the thinnest connection, suggesting that this administration route remains an exploratory regimen characterized by a small sample size and a paucity of direct comparisons within the current clinical evidence base.

To quantify the specific effects of different administration routes on reducing TSS, we generated a forest plot utilizing a random-effects model, with placebo as the reference. Based on the absolute effect sizes of the point estimates (MD), all three administration routes demonstrated a beneficial trend in ameliorating TSS symptoms (all MD values < 0). Notably, sequential ALA therapy exhibited the most substantial potential for symptom score reduction, yielding an MD of −1.75, followed by oral ALA (MD = −1.54) and IV ALA (MD = −1.14).

However, a robustness analysis incorporating 95% confidence intervals (CIs) revealed a deeper characteristic of the clinical evidence: oral ALA was the sole intervention that achieved a statistically significant difference compared to placebo (MD = −1.54, 95% CI [−3.04 to −0.04]). Because its 95% CI did not cross the line of no effect, combined with its maximum node area in the network plot, these findings demonstrate that oral administration is not only definitively efficacious in improving the TSS but also possesses the highest level of evidence robustness.

**Figure 3:**
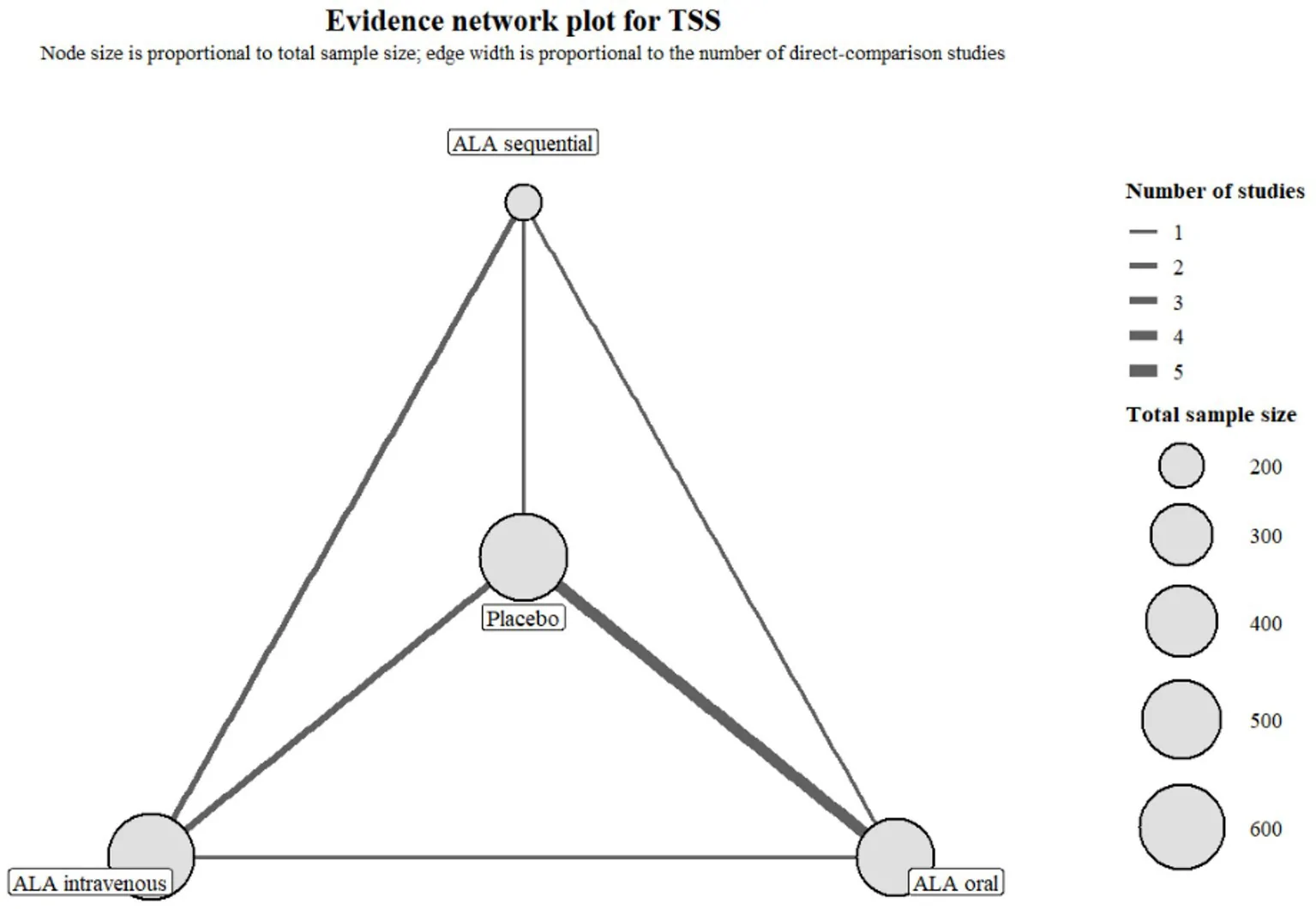
Network evidence plots for TSS.

**Figure 4:**
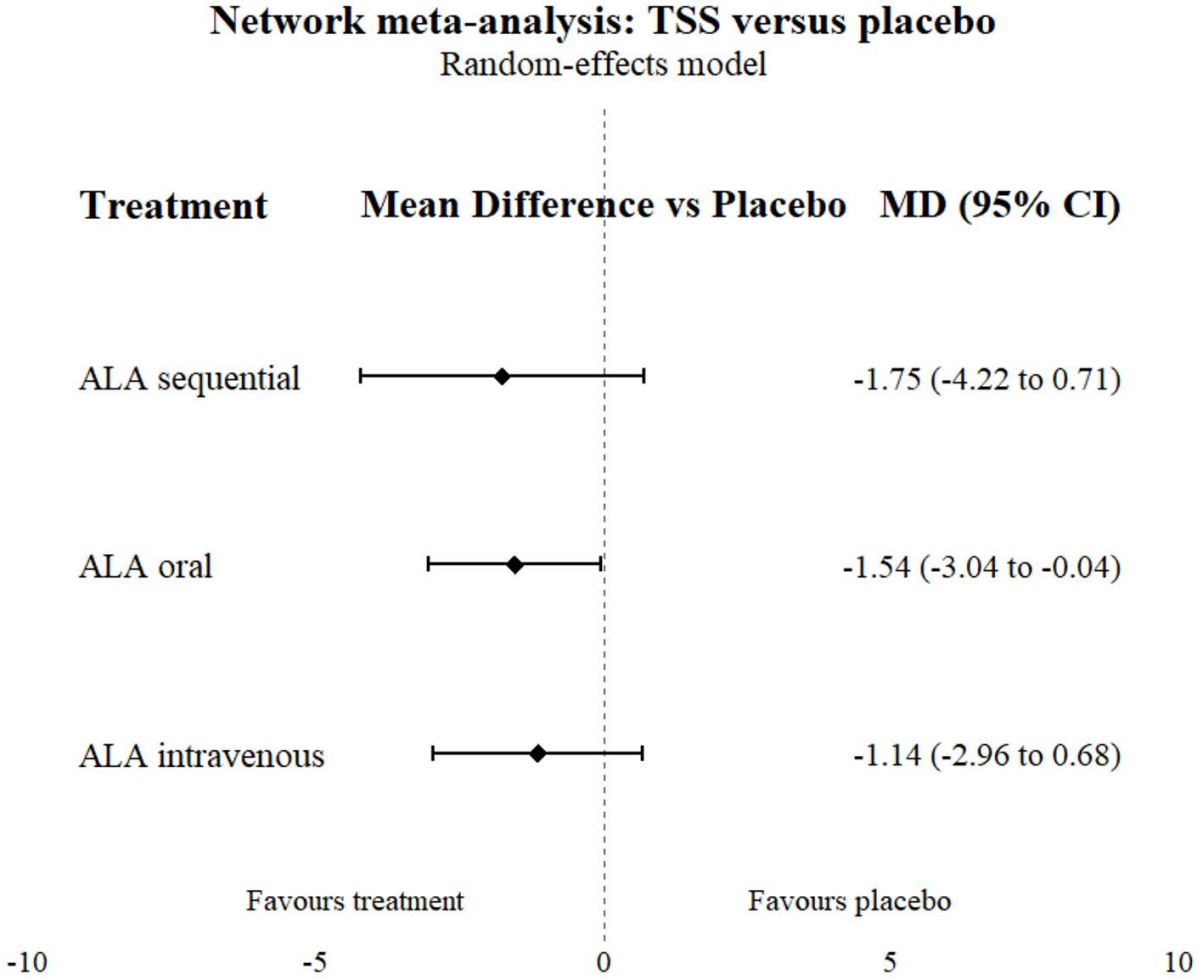
Pairwise meta-analysis for TSS.

In contrast, although sequential ALA therapy yielded the largest point estimate for benefit (−1.75), its small sample size resulted in an exceedingly wide confidence interval (95% CI [−4.22 to 0.71]) that crossed the line of no effect, thereby failing to demonstrate statistical significance in the current network model. Similarly, the pooled effect of IV ALA compared to placebo also exhibited a wide confidence interval (MD = −1.14, 95% CI [−2.96 to 0.68]). To rigorously validate these findings, we conducted a sensitivity analysis by excluding four RCTs with a high risk of bias (12, 13, 16, 20). Consequently, the sequential ALA therapy node was eliminated from the network, retaining only three nodes: placebo, oral ALA, and intravenous ALA. The Network Evidence Plots and Pairwise Meta-Analysis prior to the exclusion of these high-bias studies are detailed in Figures 3, 4.

#### 3.2.2 NIS

For the NIS, a direct evidence network comprising placebo and the three administration routes was constructed. Direct comparisons existed between IV ALA, sequential ALA therapy, and placebo; conversely, oral ALA possessed direct comparative evidence solely from comparisons with placebo, encompassing two studies. Regarding the distribution of node sizes, the placebo group constituted the largest proportion, the sample sizes for the IV ALA and oral ALA nodes were balanced, and the sequential ALA therapy node was the smallest.

**Figure 5:**
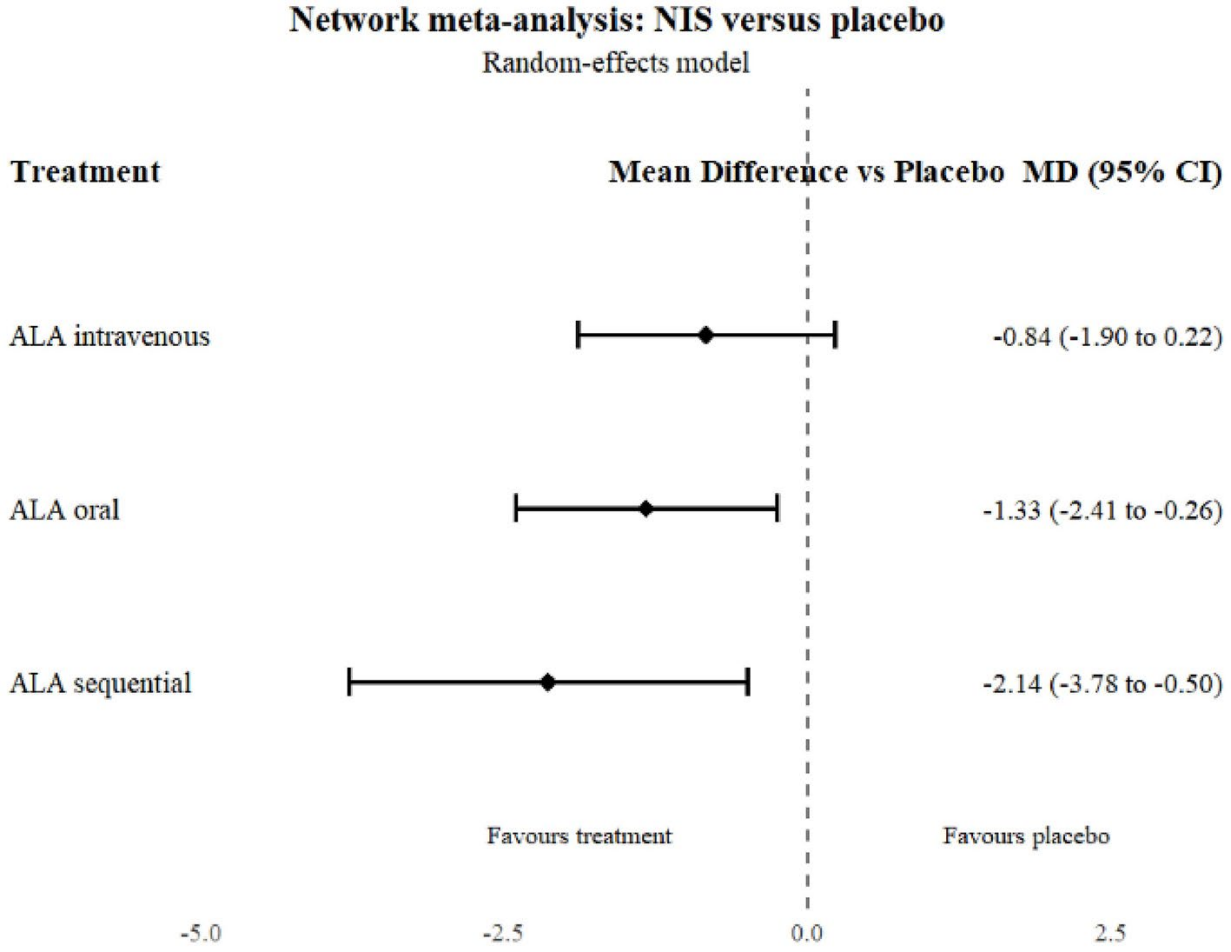
The network evidence plots for NIS.

**Figure 6:**
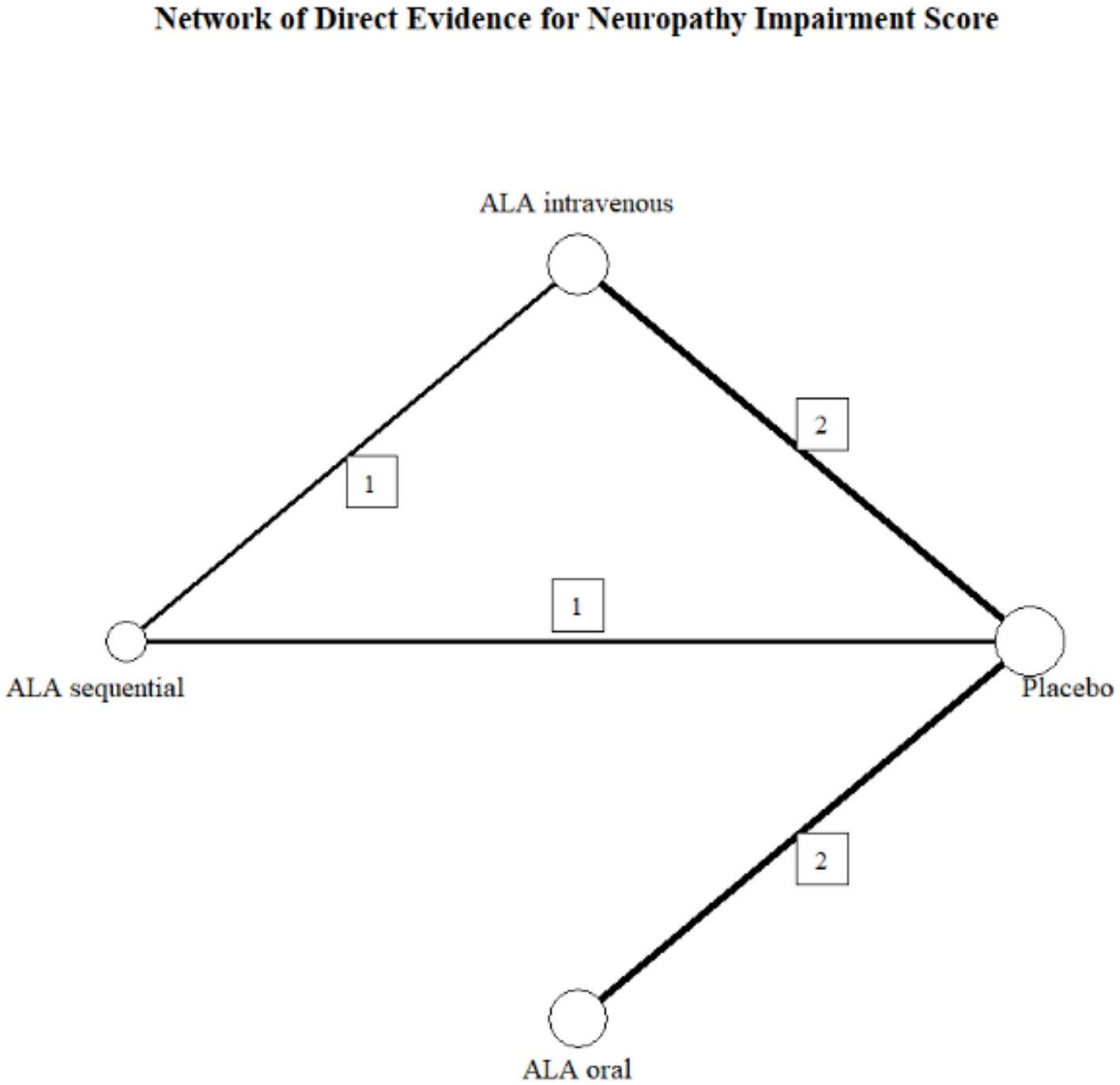
The pairwise meta-analysis for NIS.

The forest plot generated from the random-effects model revealed that, compared to placebo, sequential ALA therapy not only demonstrated the largest absolute beneficial effect size (MD = −2.14) but also achieved statistical significance, with its 95% CI situated entirely to the left of the line of no effect (95% CI [−3.78 to −0.50]). The oral ALA group likewise exhibited robust and significant efficacy, ranking second with an MD of −1.33 (95% CI [−2.41 to −0.26]). Although standalone IV ALA presented an improving trend (MD = −0.84), its 95% CI crossed the line of no effect (95% CI [−1.90 to 0.22]), failing to demonstrate a statistically significant, independent improvement on the NIS within this network model. However, the sensitivity analysis excluding the trial with a high risk of bias (13) removed the sequential ALA therapy node, refining the network to three nodes. The Network Evidence Plots and Pairwise Meta-Analysis prior to this refinement are detailed in Figures 5, 6.

#### 3.2.3 NISLL

The evidence network for the Neuropathy Impairment Score in the Lower Limbs (NIS-LL) outcome consisted of only three rigorous RCTs, with placebo serving as the core hub connecting the various interventions. Among these, oral ALA, supported by two direct comparative studies, formed the thickest edge in the network and possessed the second-largest sample node size following placebo. Comparatively, IV ALA and sequential ALA therapy relied on a single three-arm study to form a localized, closed-loop triangular network with placebo (all inter-node edge weights equaled 1), with sequential therapy representing the smallest sample node.

**Figure 7:**
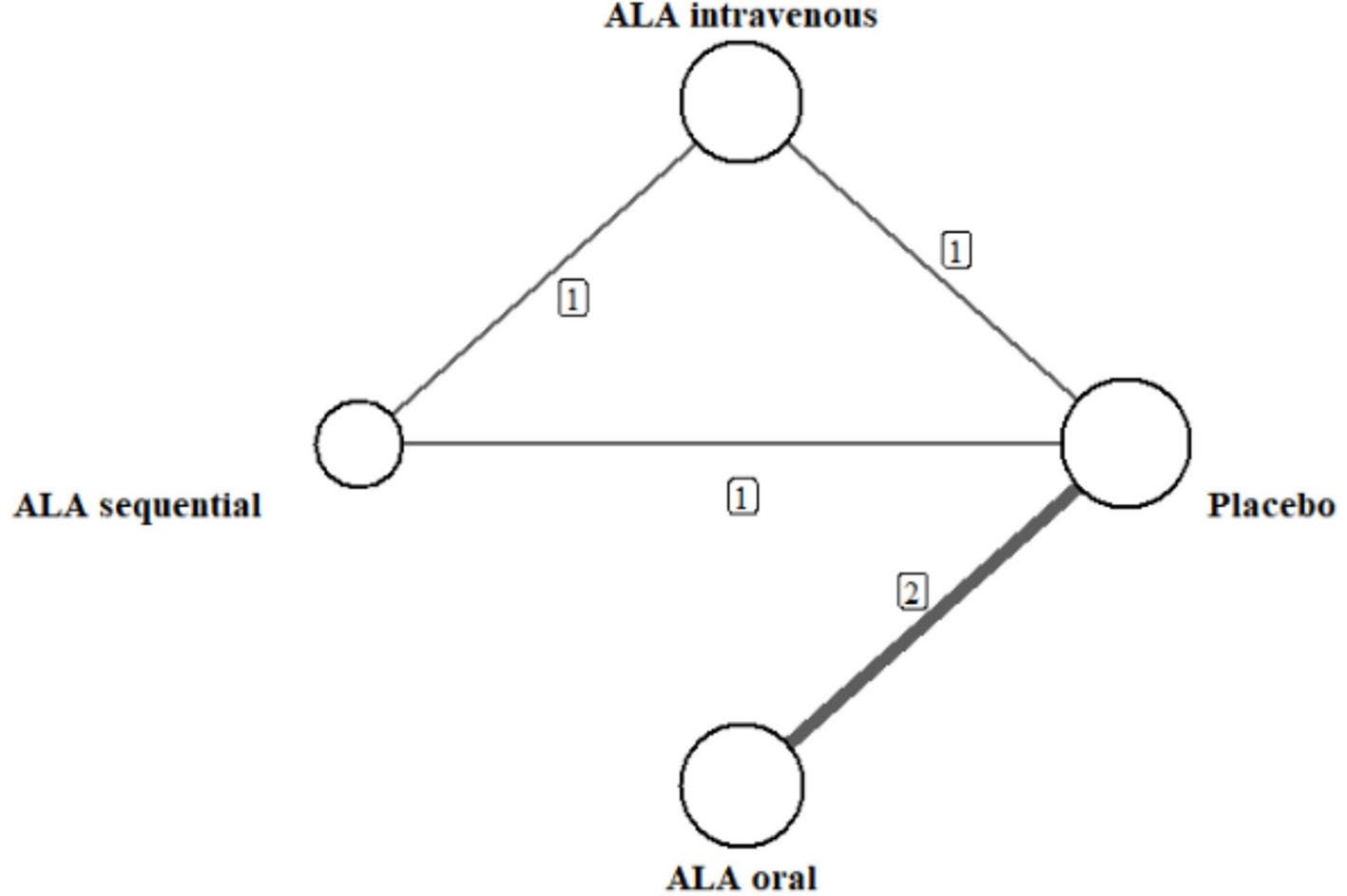
The network evidence plots for NISLL.

**Figure 8:**
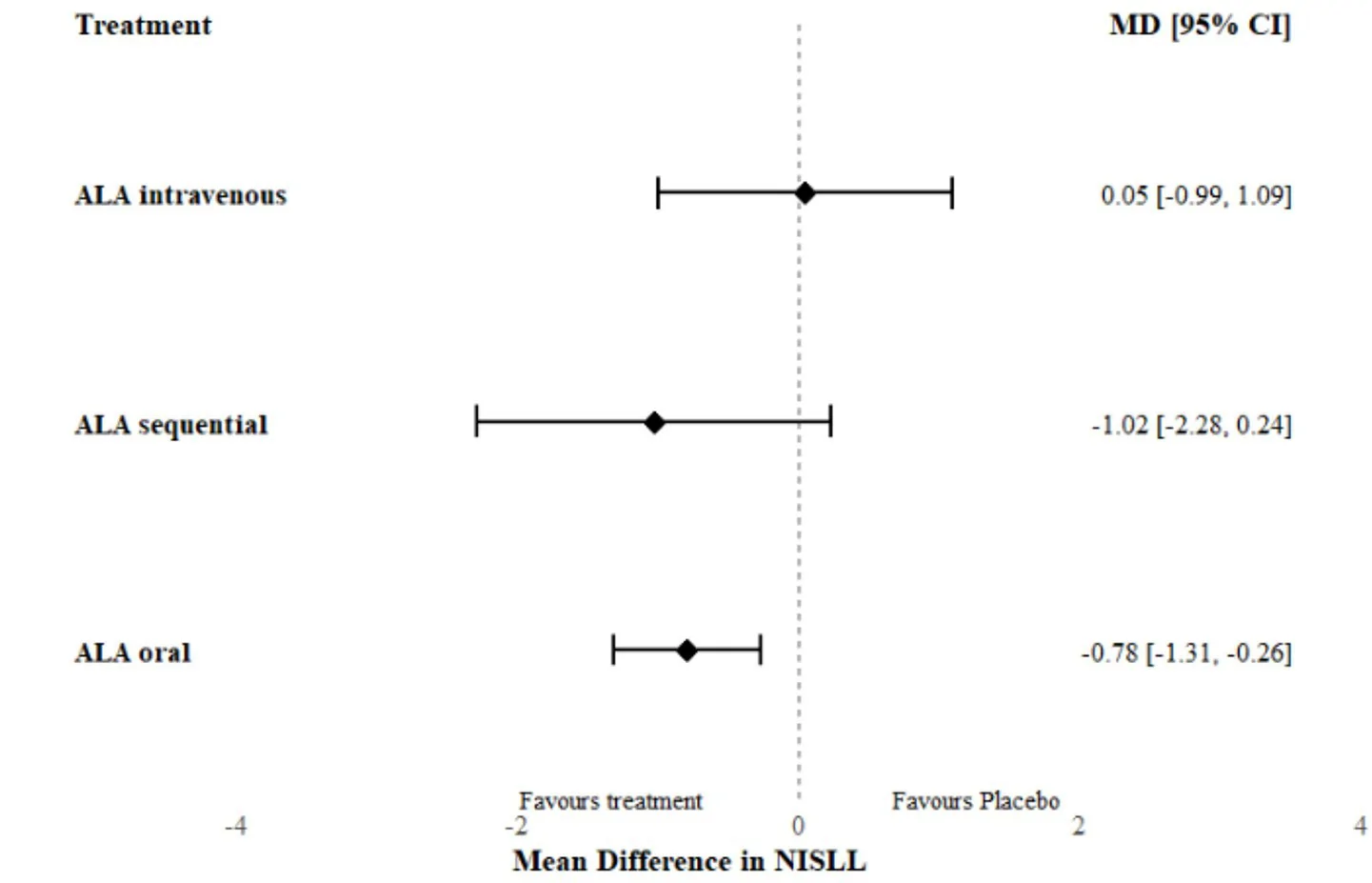
The pairwise meta-analysis for NISLL.

The forest plot indicated that oral ALA therapy was the only intervention conferring a statistically significant benefit, yielding an MD of −0.78 relative to placebo, with its 95% CI residing entirely to the left of the line of no effect (95% CI [−1.31 to −0.26]). Although sequential ALA therapy maintained the greatest point estimate trend for benefit (MD = −1.02), its sparse sample size resulted in a substantially widened confidence interval that crossed the line of no effect (95% CI [−2.28 to 0.24]), thus failing to attain statistical significance. Conversely, standalone IV ALA demonstrated virtually no effect (MD = 0.05, 95% CI [−0.99 to 1.09]). Notably, the sensitivity analysis excluding the study with a high risk of bias (13) resulted in a structural ‘collapse’ of the evidence network. With the elimination of both the intravenous and sequential therapy nodes, the network was distilled into a sole direct pairwise comparison between oral ALA and placebo. The Network Evidence Plots and Pairwise Meta-Analysis prior to the exclusion of this high-bias study are detailed in Figures 7, 8.

#### 3.2.4 GS

The GS outcome formed a V-shaped open, sparse network anchoring three nodes: IV ALA, oral ALA, and placebo. Direct head-to-head comparative evidence between the IV and oral ALA groups was absent. Edge weights indicated that the IV administration group was supported by two direct comparative studies, whereas the oral group had only one; furthermore, the sample sizes of the IV administration and placebo nodes were substantially larger than that of the oral group. Due to the sparsity of the network and the absence of closed loops, consistency testing was omitted for this outcome.

**Figure 9:**
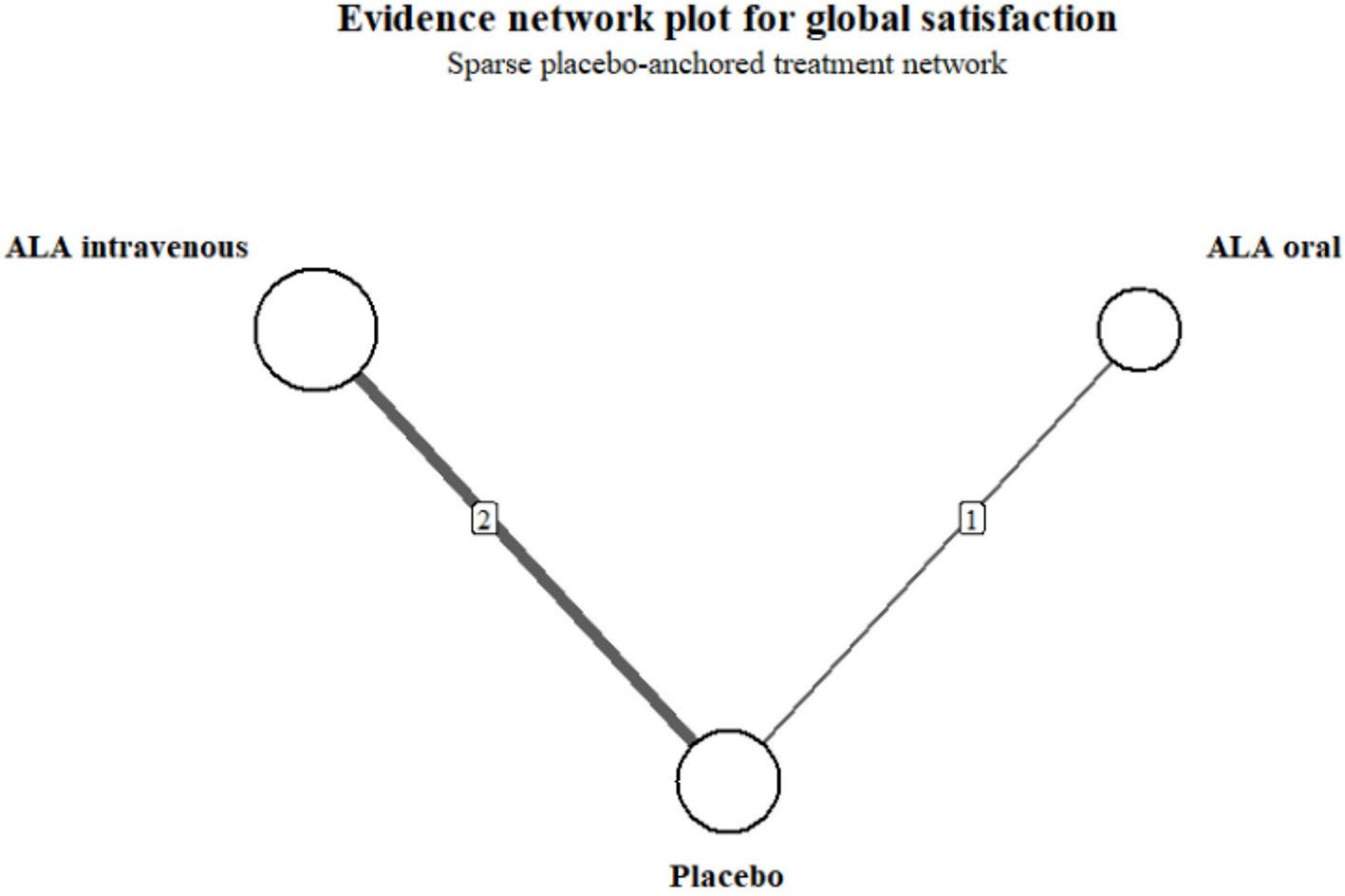
The relevant network evidence plots for GS.

**Figure 10:**
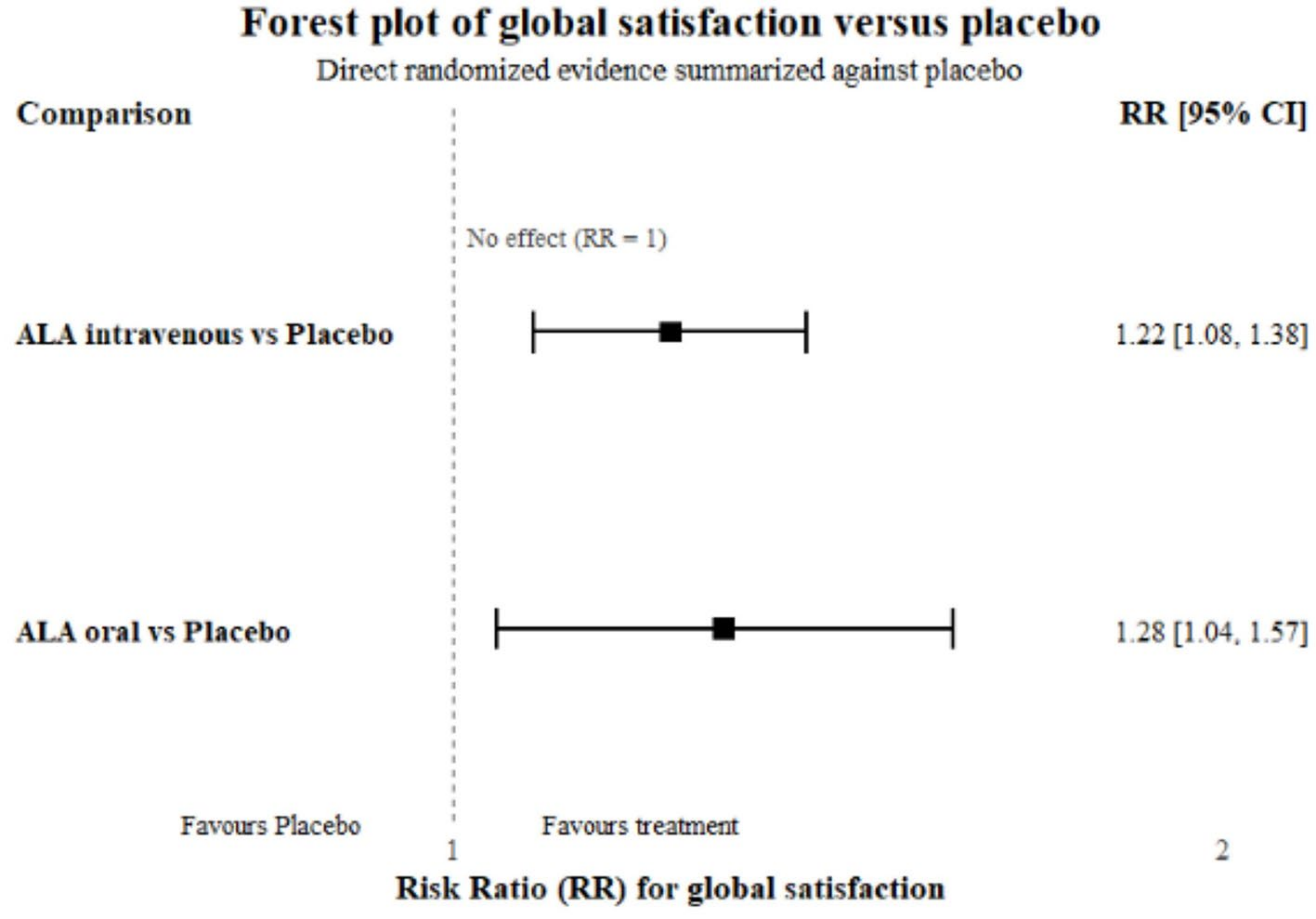
The pairwise meta-analysis for GS.

Given that GS is a categorical variable, we utilized the risk ratio (RR) rather than the mean difference (MD to quantify its effect size). Contrary to the “lower is better” logic applied to scoring scales, an RR value greater than 1 for satisfaction denotes that the intervention is superior to placebo. The forest plot illustrated that, despite the sparse evidence network, both available standalone ALA routes significantly enhanced global satisfaction. Oral ALA therapy exhibited a higher point estimate for satisfaction benefit compared to placebo (RR = 1.28), and its 95% CI resided entirely to the right of the line of no effect (95% CI [1.04 to 1.57]), achieving statistical significance. Similarly, IV ALA demonstrated a consistent and significant positive effect (RR = 1.22, 95% CI [1.08 to 1.38]); moreover, owing to its larger sample size, its confidence interval was narrower and more robust. The relevant Network Evidence Plots and Pairwise Meta-Analysis is detailed into Figures 9, 10.

### 3.3 Network meta-analysis results and SUCRA rankings

#### 3.3.1 TSS

The upper-right quadrant of the pairwise comparison league table for the TSS displays the direct comparative evidence derived from conventional pairwise meta-analyses, whereas the lower-left quadrant presents the pooled effect sizes from the NMA, integrating both direct and indirect evidence. According to the pooled NMA results, with placebo serving as the common reference, all three ALA administration routes demonstrated a trend toward reducing TSS (all MD < 0).

Oral ALA therapy was the sole intervention to achieve a statistically significant difference compared to placebo (MD = −1.54, 95% CI [−3.04 to −0.04]). Although sequential ALA therapy exhibited the greatest benefit in absolute effect size (MD = −1.75), its wide confidence interval crossed the line of no effect (95% CI [−4.22 to 0.71]). Head-to-head comparisons among the active interventions (e.g., oral ALA vs. IV ALA: MD = 0.40, 95% CI [−1.72 to 2.51]) did not reveal any statistically significant differences.

To evaluate the probability of each intervention being the optimal treatment regimen, the surface under the cumulative ranking curve (SUCRA) was calculated. The SUCRA rankings were highly consistent with the effect size trends observed in the league table. Sequential ALA therapy ranked first (SUCRA: 72.4%), emerging as the route with the greatest potential for improving TSS. Oral ALA therapy ranked second (68.2%), IV ALA therapy ranked third (51.7%), and the placebo group ranked last (7.6%).

However, in evaluating global heterogeneity, the Bayesian network meta-analysis yielded a between-study variance τ2 of 2.95 (95% CrI: 1.08 to 10.13) for the TSS outcome. Pairwise meta-analyses for direct comparisons revealed substantial heterogeneity, as expected given the varying clinical protocols. Specifically, the I^2^ for oral ALA versus placebo (5 trials) was 85.5% (Cochran’s Q = 27.57, *p* < 0.001), and for intravenous ALA versus placebo (3 trials) was 97.4% (Cochran’s Q = 78.38, p < 0.001).

**Figure 11:**
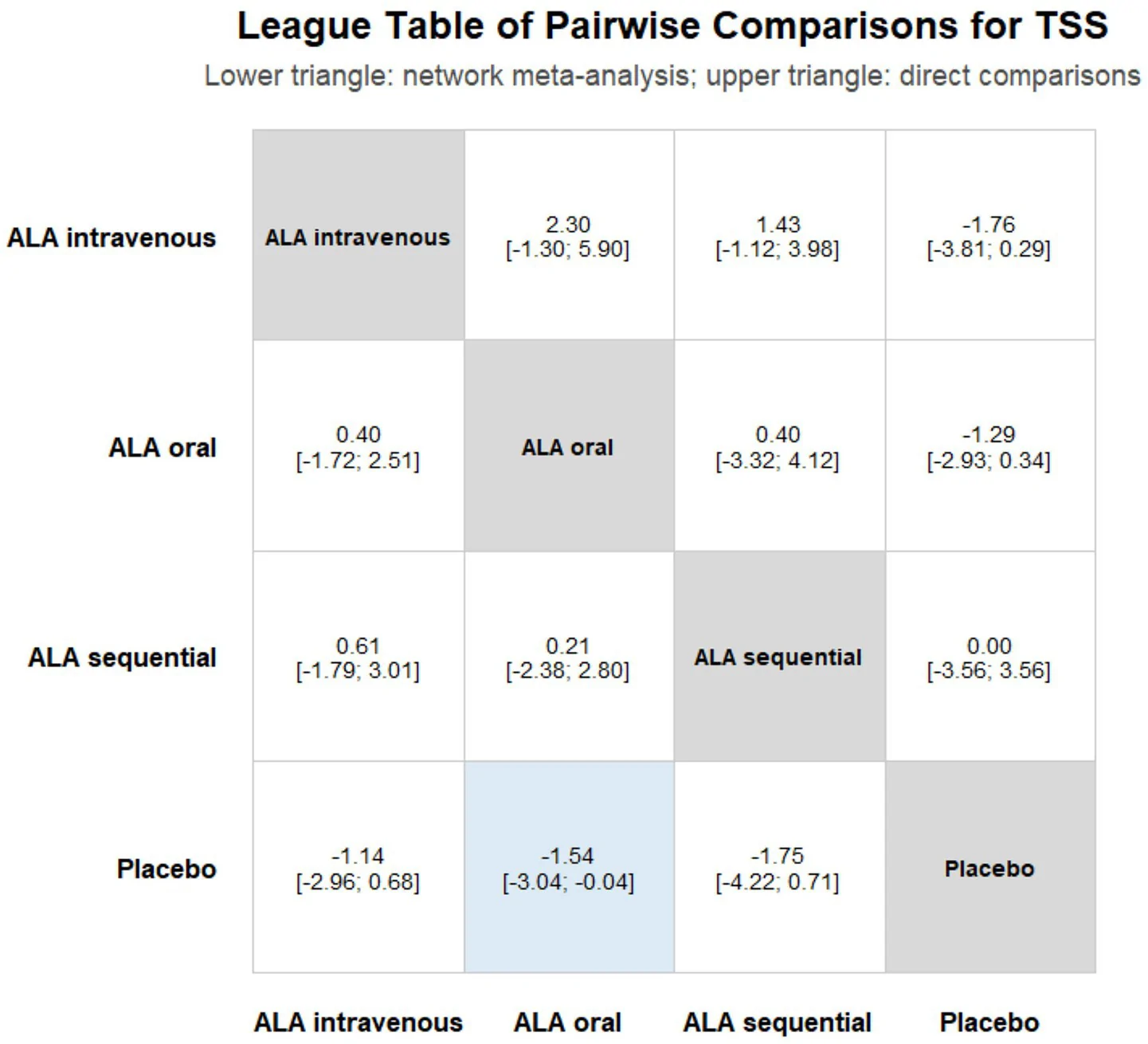
The network meta-analysis results for TSS.

**Figure 12:**
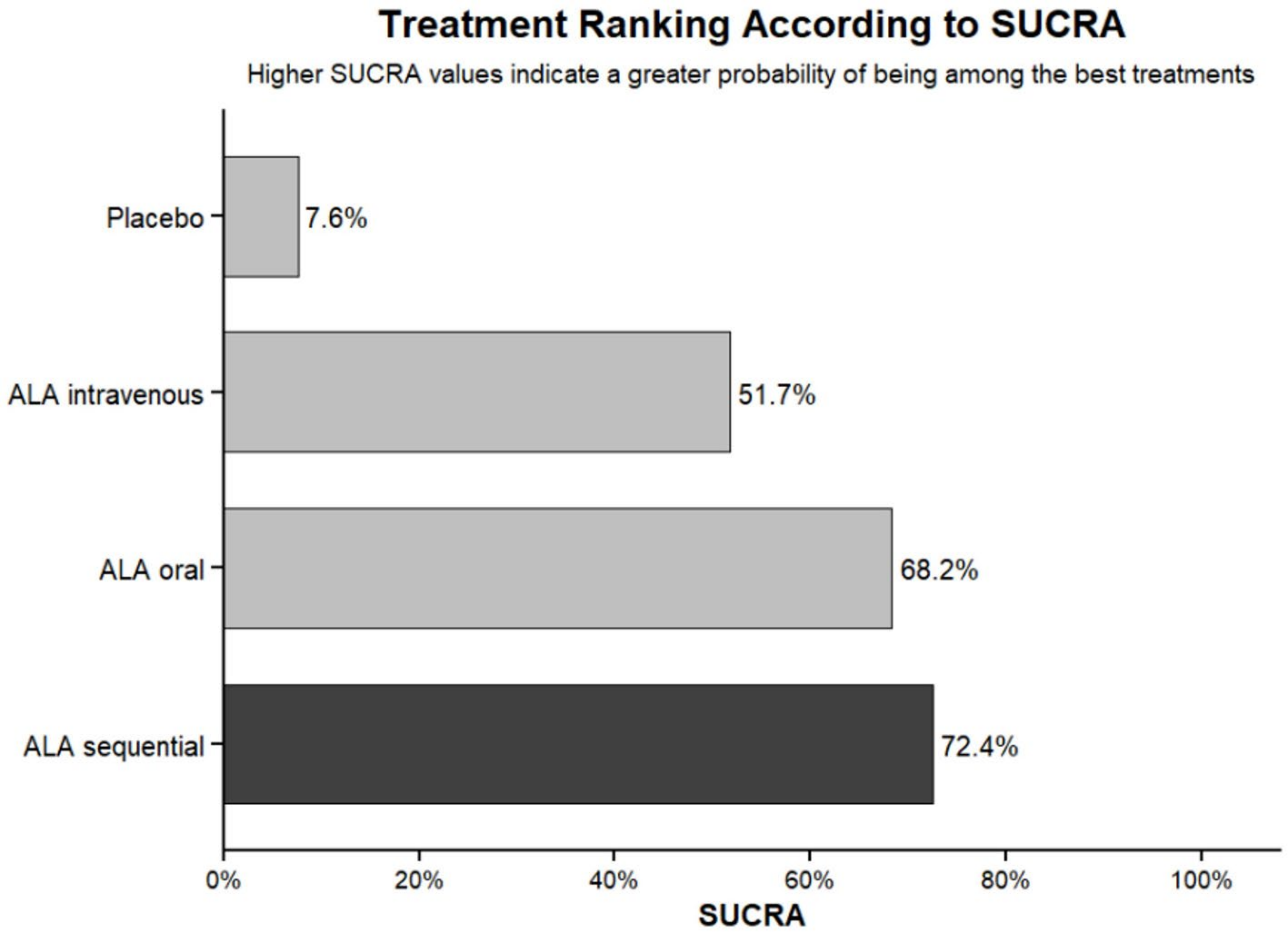
The SUCRA rankings for TSS.

Accordingly, we excluded the studies with a high risk of bias. Upon the removal of the sequential therapy node, intravenous therapy unmasked its potent efficacy, becoming highly significant compared to placebo (MD = −2.81, 95% CI [−4.70 to −0.92]) and securing the top rank (SUCRA: 85.3%). Conversely, oral ALA experienced an attenuation in effect size and lost its statistical significance (MD = −1.12, 95% CI [−2.70 to 0.47]), dropping to the second rank (SUCRA: 14.1%). The Network Meta-Analysis Results and SUCRA Rankings prior to conducting the sensitivity analysis are detailed in Figures 11, 12.

#### 3.3.2 NIS

Within the pooled NMA results in the lower-left quadrant of the NIS league table, both sequential and oral ALA therapies achieved statistical significance compared to placebo. Sequential ALA therapy demonstrated the highest absolute efficacy (MD = −2.14, 95% CI [−3.78 to −0.50]), followed by oral ALA therapy (MD = −1.33, 95% CI [−2.41 to −0.26]). Although standalone IV ALA therapy showed an improving trend, it did not reach statistical significance (MD = −0.84, 95% CI [−1.90 to 0.22]).

Notably, in direct pairwise comparisons (upper-right quadrant), sequential ALA therapy did not reach statistical significance compared to placebo (MD = −1.45, 95% CI [−3.35 to 0.45]). However, in the direct head-to-head comparison, sequential ALA therapy was significantly superior to standalone IV ALA therapy (MD = 1.48, 95% CI [0.04 to 2.92]). This pivotal direct comparative evidence enabled sequential therapy to narrow its confidence interval within the network framework, ultimately confirming its significant efficacy in improving the NIS.

In the SUCRA results, sequential ALA therapy secured the top rank (85.5%), while oral ALA therapy ranked second (65.2%). Potentially due to its inability to provide sustained, long-term neural repair, the ranking of standalone IV ALA therapy dropped substantially to third place (41.9%), with placebo ranking last (7.4%).

In evaluating global heterogeneity across the network, the Bayesian between-study variance (τ2) was estimated at 0.41 (95% CrI: 0.00 to 2.08) for the objective NIS outcome. Traditional pairwise meta-analyses further confirmed this statistical stability. Most strikingly, the comparison between oral ALA and placebo for NIS demonstrated complete homogeneity (I^2^ = 0.0%, Cochran’s Q = 0.018, *p* = 0.89), while moderate heterogeneity was observed in the intravenous ALA vs. placebo group (I^2^ = 52.7%, *p* = 0.15).

**Figure 13:**
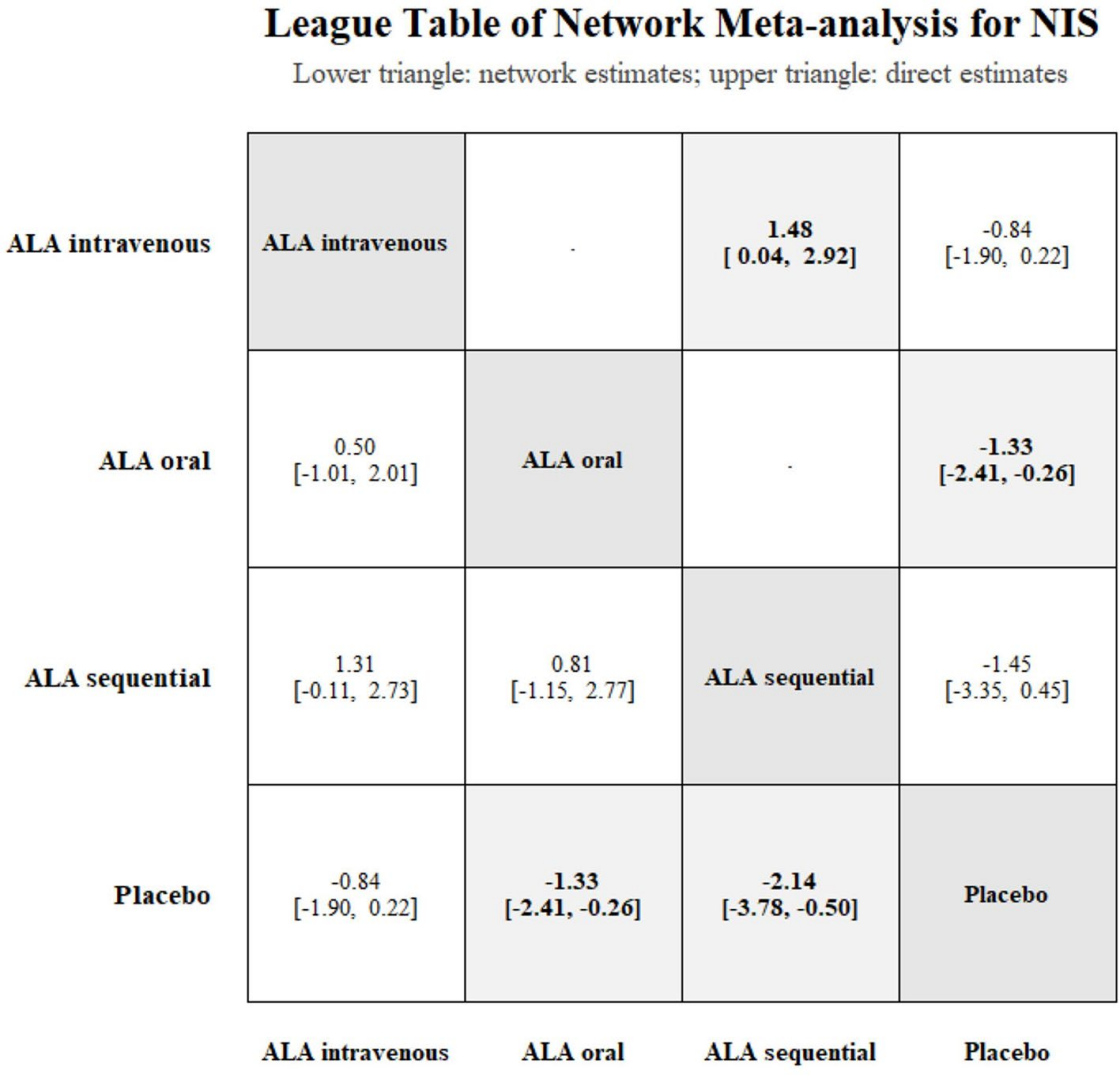
The network meta-analysis results for NIS.

**Figure 14:**
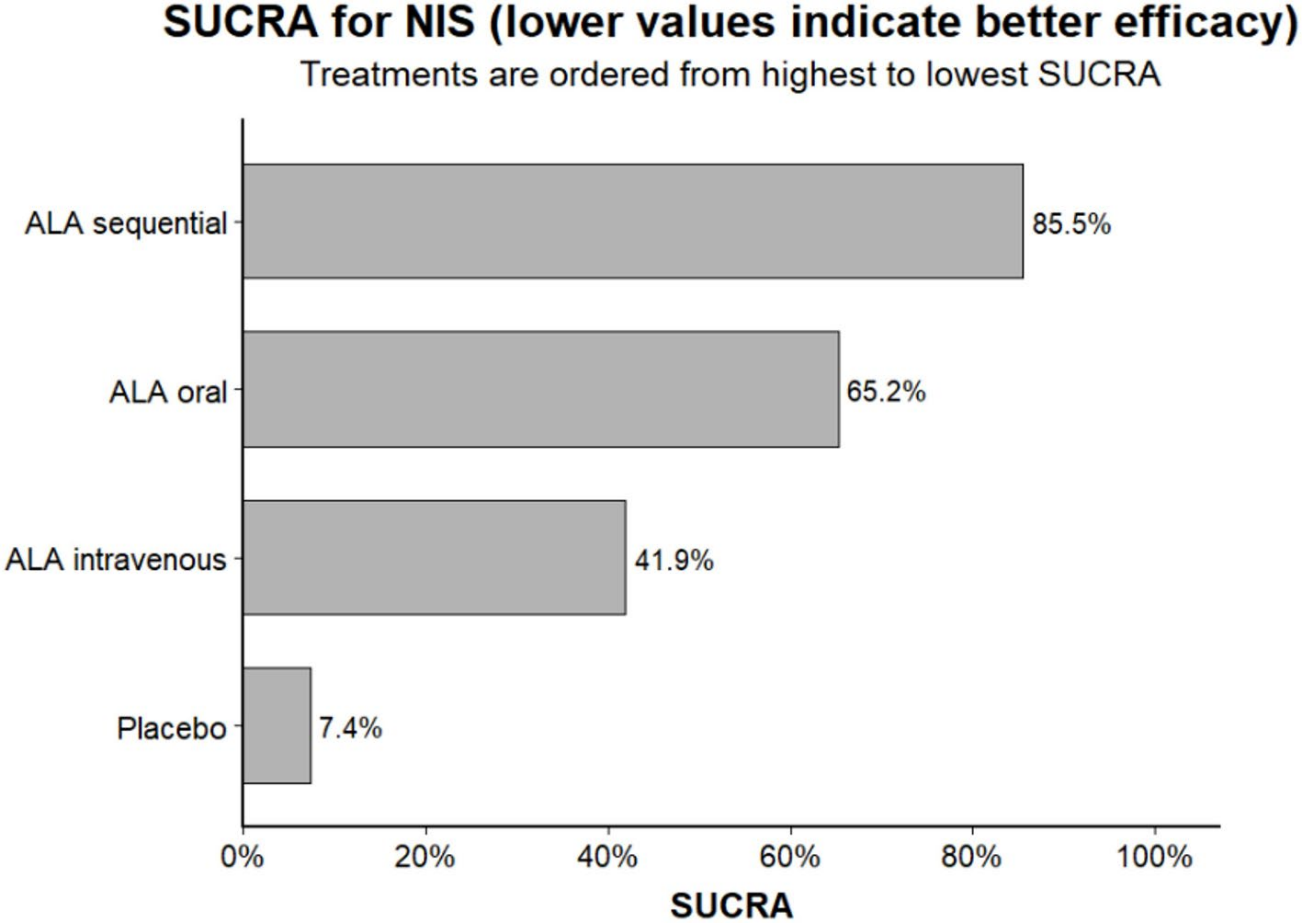
The SUCRA rankings for NIS.

Subsequently, upon conducting the sensitivity analysis, the removal of the sequential therapy node allowed intravenous therapy to transition from non-significant to statistically significant (MD = −1.50, 95% CI [−2.85 to −0.15]). It leaped to the first rank (SUCRA: 41.7%), closely tied with the consistently robust oral ALA therapy (MD = −1.33, 95% CI [−2.36 to −0.30]; SUCRA: 40.8%). The Network Meta-Analysis Results and SUCRA Rankings prior to the sensitivity analysis are detailed in Figures 13, 14.

#### 3.3.3 NISLL

DPN is typically characterized by length-dependent distal involvement, making the NIS-LL—which reflects objective signs in the lower extremities—the most refractory endpoint to reverse. The NMA results (lower-left quadrant) revealed that oral ALA therapy was the only intervention to demonstrate a statistically significant benefit compared to placebo (MD = −0.78, 95% CI [−1.31 to −0.26]).

In the pooled network comparisons, sequential ALA therapy was significantly superior to standalone IV ALA therapy (MD = −1.07, 95% CI [−2.05 to −0.09]), a finding corroborated by the direct head-to-head comparison (upper-right quadrant). Standalone IV ALA therapy lacked statistical significance in the pooled network assessment, and its point estimate even exhibited a directional reversal (MD = 0.05 vs. placebo). This demonstrates that short-term intravenous administration fails to provide effective support for the substantial repair of distal lower-extremity nerves.

SUCRA results demonstrated that sequential ALA therapy dominated the first rank (81.4%), closely followed by oral ALA therapy (73.0%); together, they constituted the top tier for lower-extremity nerve repair. Conversely, the ranking for IV ALA therapy declined sharply (22.9%), placing it in nearly the same echelon as the ineffective placebo group (22.6%).

To adequately explore global and local heterogeneity, both Bayesian between-study variance (τ2) and frequentist pairwise I^2^ statistics were calculated. Strikingly, heterogeneity was virtually eliminated when assessing the most refractory distal impairment metric (NIS-LL), which yielded an exceptionally low network τ2 of 0.14 (95% CrI: 0.00 to 1.05) and complete homogeneity in the oral vs. placebo direct comparison (I^2^ = 0.0%, Cochran’s Q = 0.001, *p* = 0.97). Furthermore, direct comparative evidence for several advanced regimens (e.g., sequential vs. placebo) in NIS-LL was limited to single trials, powerfully underscoring the absolute necessity of our NMA framework to integrate these disconnected nodes.

**Figure 15:**
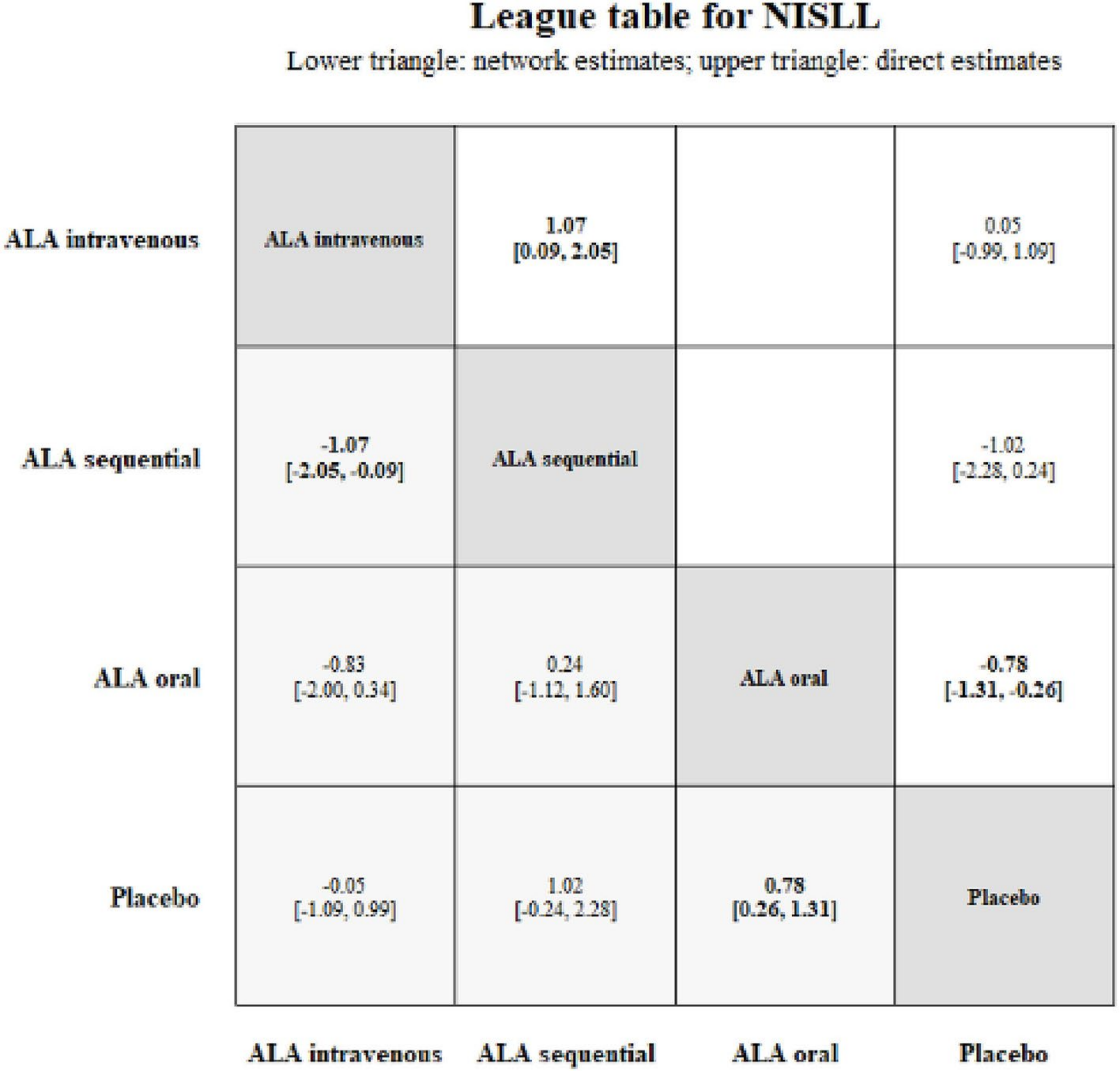
The network meta-analysis results for NISLL.

**Figure 16:**
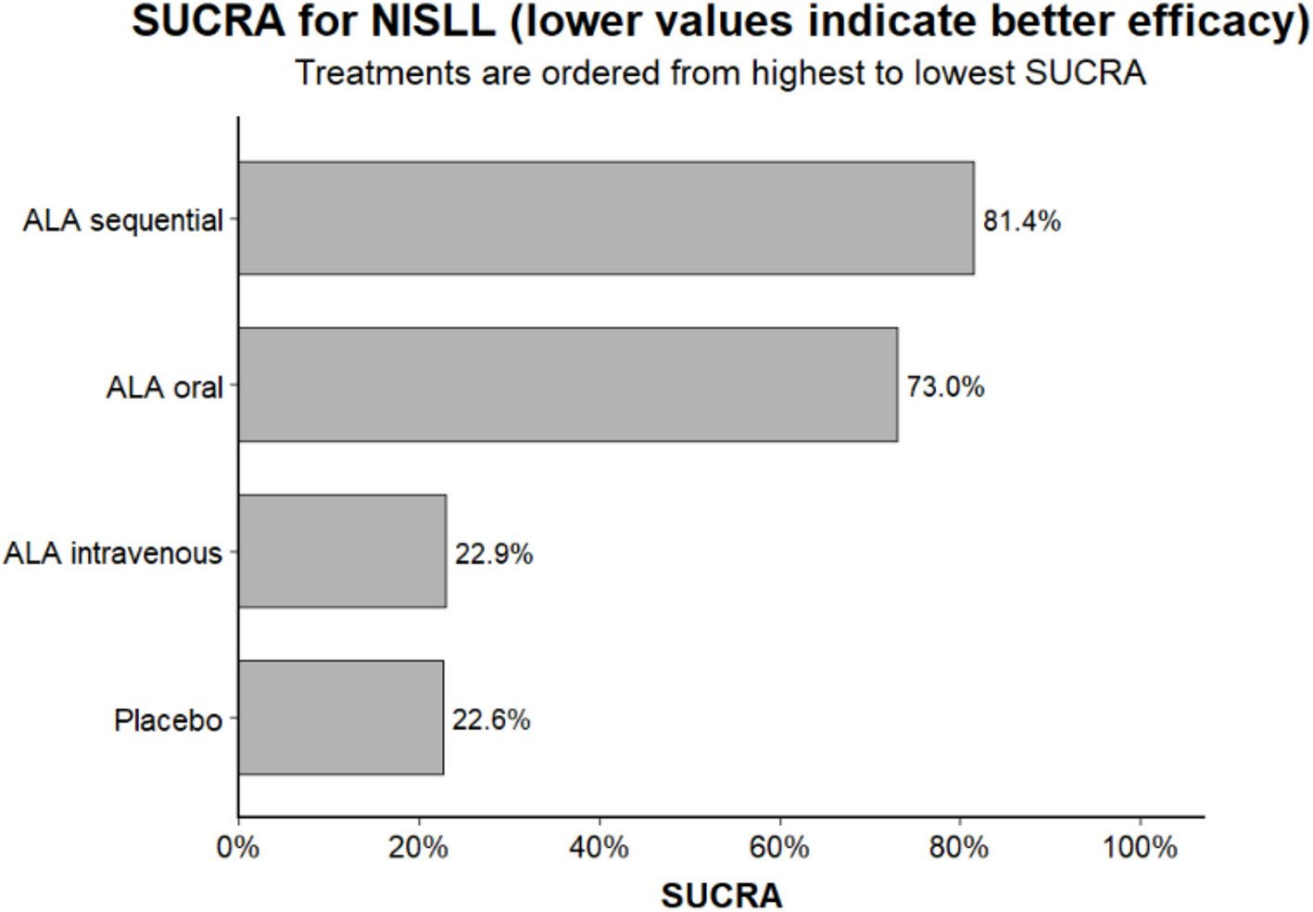
The SUCRA rankings for NISLL.

Subsequently, the sensitivity analysis was executed. With the IV and sequential therapies eliminated from the unbiased network, oral ALA stood alone as the sole intervention supported by robust, high-quality evidence demonstrating significant efficacy (MD = −0.78, 95% CI [−1.31 to −0.26]), decisively outperforming placebo with an overwhelming 97.4% probability of being the optimal treatment. The Network Meta-Analysis Results and SUCRA Rankings prior to the exclusion of these therapies are detailed in Figures 15, 16.

#### 3.3.4 GS

Due to the lack of closed loops in the evidence network for the GS outcome, we constructed a league table exclusively presenting the pooled network effect sizes (lower-left matrix), utilizing the RR as the metric. The RR for placebo versus oral ALA therapy was 0.78 (95% CI [0.63 to 0.98]), and versus IV ALA therapy was 0.82 (95% CI [0.73 to 0.93]). Because statistical significance in this matrix format indicates that an RR < 1 favors the active intervention over placebo, both oral and intravenous ALA administrations effectively elevated global treatment satisfaction.

**Figure 17:**
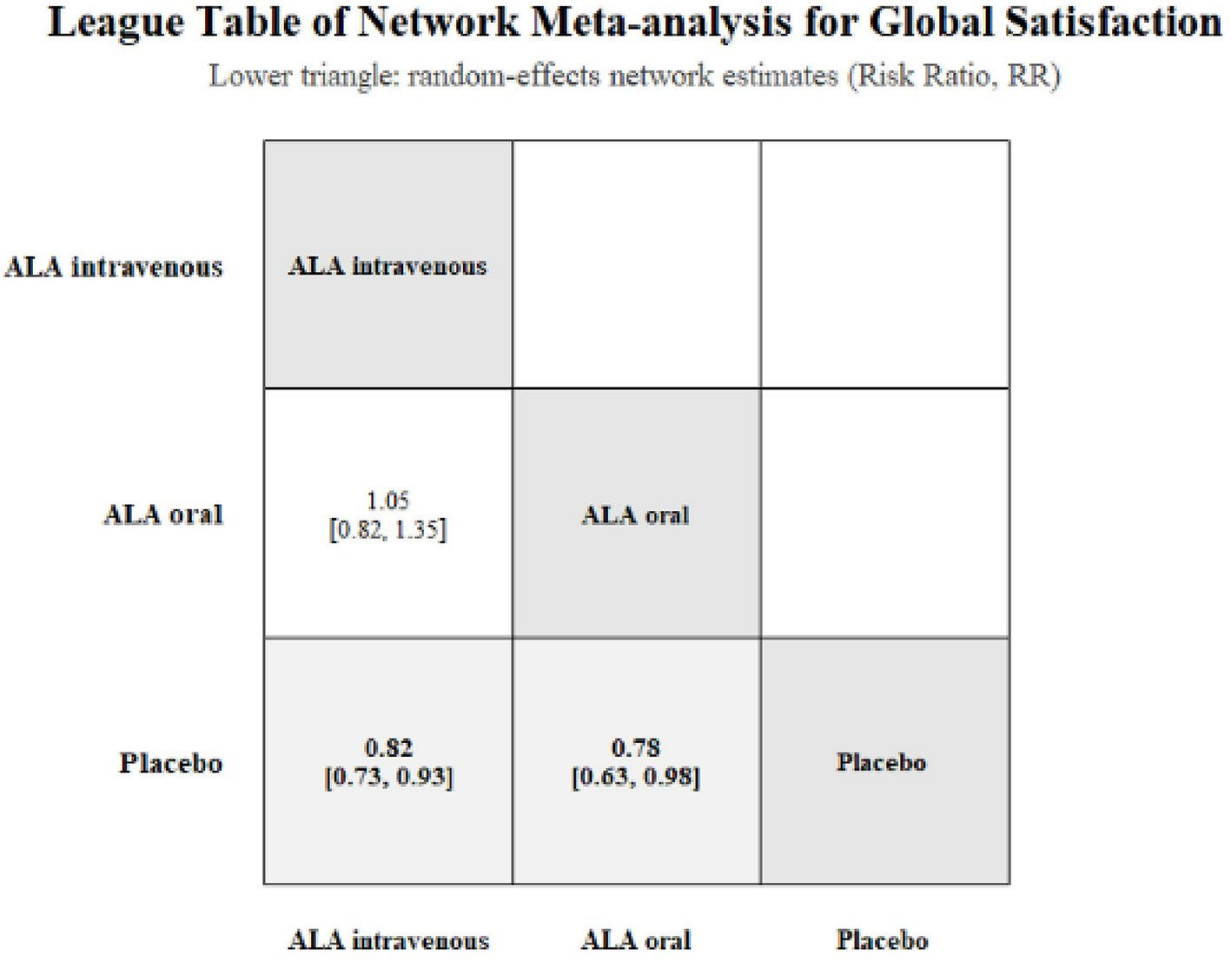
The network meta-analysis results for GS.

Oral ALA therapy exhibited a slight point estimate advantage compared to IV ALA therapy (RR = 1.05). However, its 95% CI was wide and crossed the line of no effect (95% CI [0.82 to 1.35]), failing to reach statistical significance. These findings suggest that oral administration, by virtue of its point estimate advantage and the clinical convenience of avoiding inpatient infusions, demonstrates a potential clinical benefit that is non-inferior—and perhaps slightly superior—to standalone intravenous therapy. The relevant Network Meta-Analysis Results and SUCRA Rankings is detailed in Figure 17.

The evidence network for global satisfaction was open, consisting of comparisons between intravenous ALA and placebo and between oral ALA and placebo, with no closed loops; therefore, inconsistency could not be assessed. The frequentist network meta-analysis estimated a low level of between-study heterogeneity across the network (*τ* = 0.0400). Because the oral ALA evidence was derived from a single trial, the observed heterogeneity originated entirely from the two intravenous ALA comparisons. A pairwise random-effects meta-analysis using the restricted maximum likelihood estimator with the Knapp–Hartung adjustment demonstrated low statistical heterogeneity (I^2^ = 11.7%, τ^2^ = 0.0016, Cochran’s Q = 1.132, *p* = 0.287), indicating good consistency between the two intravenous studies.

### 3.4 Dual-outcome quadrant plots

#### 3.4.1 TSS

To delineate the balance between clinical efficacy and potential safety risks across the various administration routes, this study constructed dual-outcome quadrant plots. In these plots, the effect estimate of the placebo serves as the origin of the Cartesian coordinate system, stratifying the overall performance of the active interventions into four distinct quadrants.

**Figure 18:**
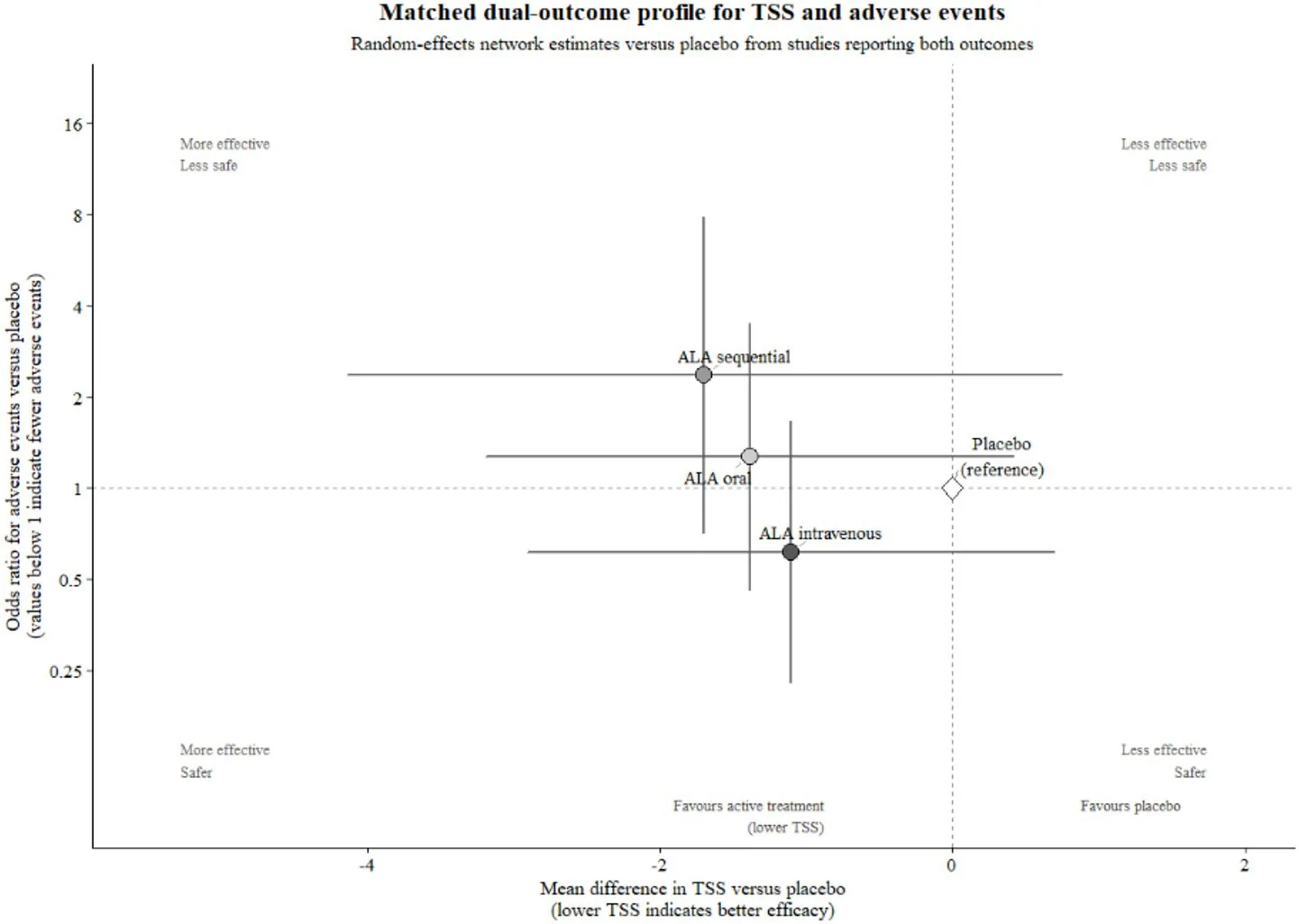
Dual-outcome quadrant plots for TSS.

In the dual-outcome plot for the TSS, a trade-off dynamic was observed between efficacy (X-axis: mean difference [MD]) and adverse events (Y-axis: odds ratio [OR]). Both oral and sequential ALA therapies were situated in the upper-left quadrant, denoting higher efficacy but a marginal increase in safety risk. Oral therapy emerged as the sole intervention statistically confirmed to significantly alleviate subjective symptoms; its OR was slightly greater than 1 but its 95% confidence interval crossed the null threshold (OR = 1), indicating that while a minor upward trend in adverse events existed, it remained within clinically acceptable limits. IV ALA therapy, yielding an OR of approximately 0.6, was located in the lower-left “dual-optimal” quadrant, suggesting a profile that is simultaneously more effective and safer than placebo. However, owing to its wide, bidirectional confidence intervals, this potential advantage failed to achieve statistical significance. Following the sensitivity analysis, IV ALA firmly anchored itself in this ‘dual-optimal’ lower-left quadrant, achieving statistical significance. The dual-outcome quadrant plots prior to the sensitivity analysis are detailed in Figure 18.

#### 3.4.2 NIS

**Figure 19:**
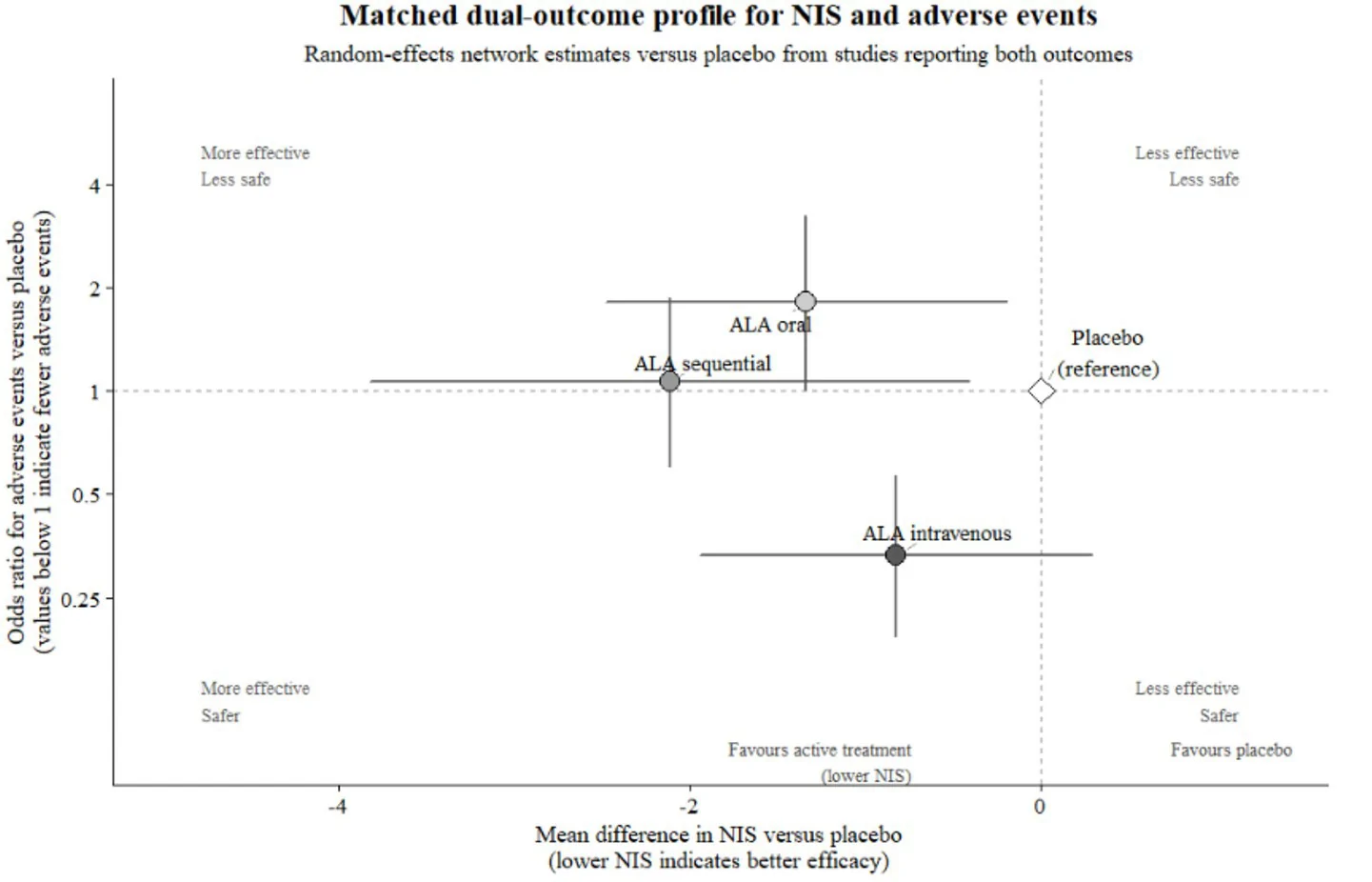
The dual-outcome quadrant plots for NIS.

For sequential ALA therapy, the MD reached −2.14 alongside an adverse event OR of approximately 1.1. Compared to its position in the TSS plot, this represented a pronounced leftward and downward shift. This positional shift signifies that, in reversing objective neurological signs, sequential therapy not only exerts the highest absolute efficacy but also incurs no additional safety penalty, thereby achieving a superior benefit–risk profile. In contrast, while oral therapy maintained its statistically significant efficacy, its safety indicator remained elevated within the upper quadrant. Upon excluding the biased data from Ziegler (13), IV ALA successfully repositioned into the ‘dual-optimal’ lower-left quadrant. It demonstrated significant objective efficacy coupled with an excellent safety profile, matching the steady performance of oral therapy. The dual-outcome quadrant plots prior to this exclusion are detailed in Figure 19.

#### 3.4.3 NISLL

**Figure 20:**
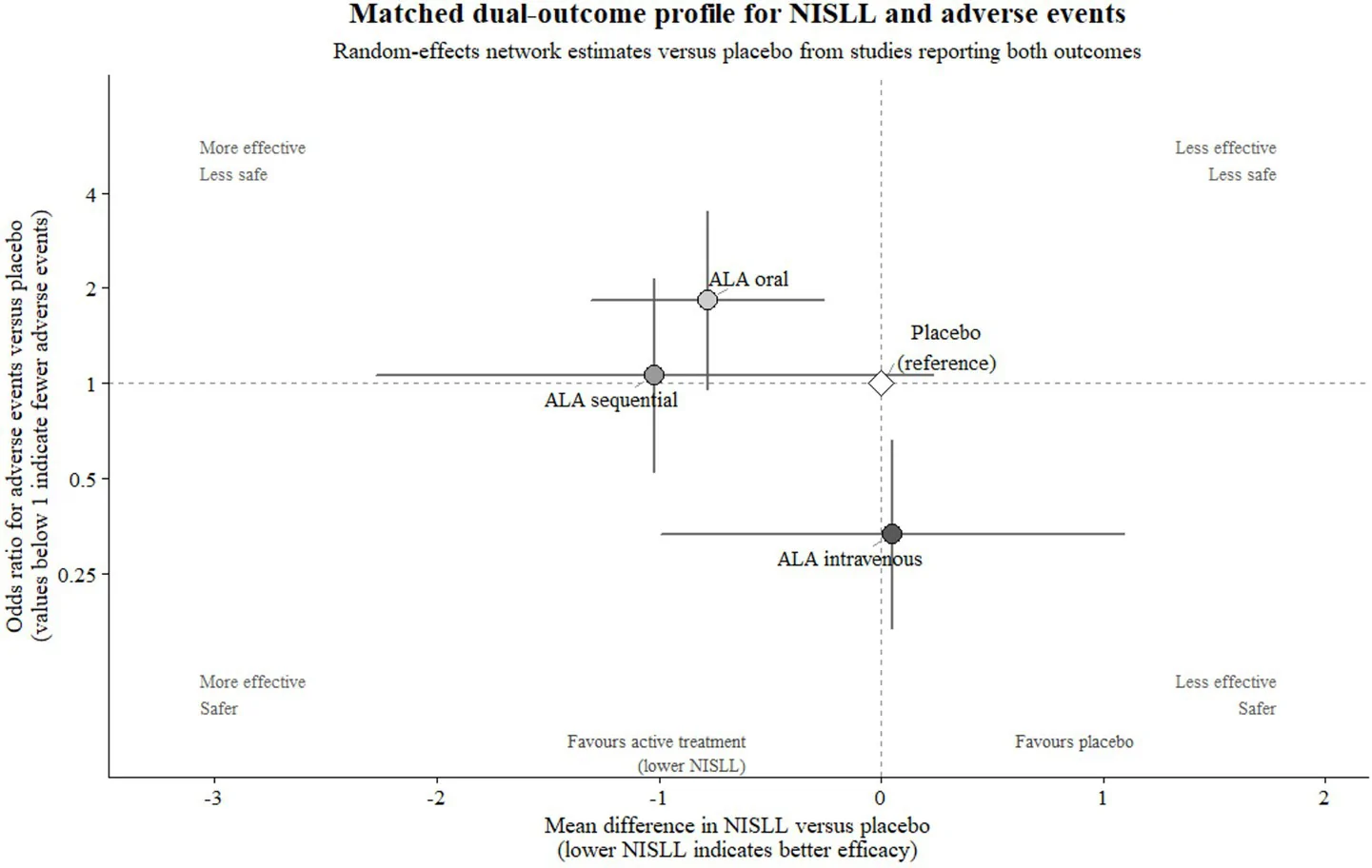
The dual-outcome quadrant plots for NISLL.

When focusing on the most refractory endpoint of DPN—distal lower extremity nerve impairment as measured by the NIS-LL—IV ALA therapy was located in the lower-right quadrant. This placement, signifying an ineffective yet safe profile, highlights the failure of short-term intravenous administration to facilitate deep nerve repair in the lower extremities. Oral ALA therapy was the only administration route firmly anchored in the effective quadrant, with its horizontal confidence interval completely clear of the null threshold. This suggests that when confronting the most recalcitrant lower-extremity signs, the enduring clinical benefits conferred by long-term oral administration are sufficient to offset the accompanying mild risk of adverse events. Following the sensitivity analysis, oral ALA remained solitary within the effective quadrant of the entire NIS-LL dual-outcome plot. This further cements its exclusive position as the safest and most effective evidence-based option for distal nerve repair. The dual-outcome quadrant plots prior to the sensitivity analysis are detailed in Figure 20.

#### 3.4.4 GS

In the dual-outcome plot for GS, the X-axis metric transitioned to the RR, where placement to the right of the vertical null line indicates superiority over placebo. The plot revealed that both the oral and intravenous routes fell into the right-hand quadrants, demonstrating significant clinical benefit. On the Y-axis, IV ALA therapy aligned closely with the horizontal baseline of the placebo, representing a highly favorable paradigm of high satisfaction with no detectable additional risk.

**Figure 21:**
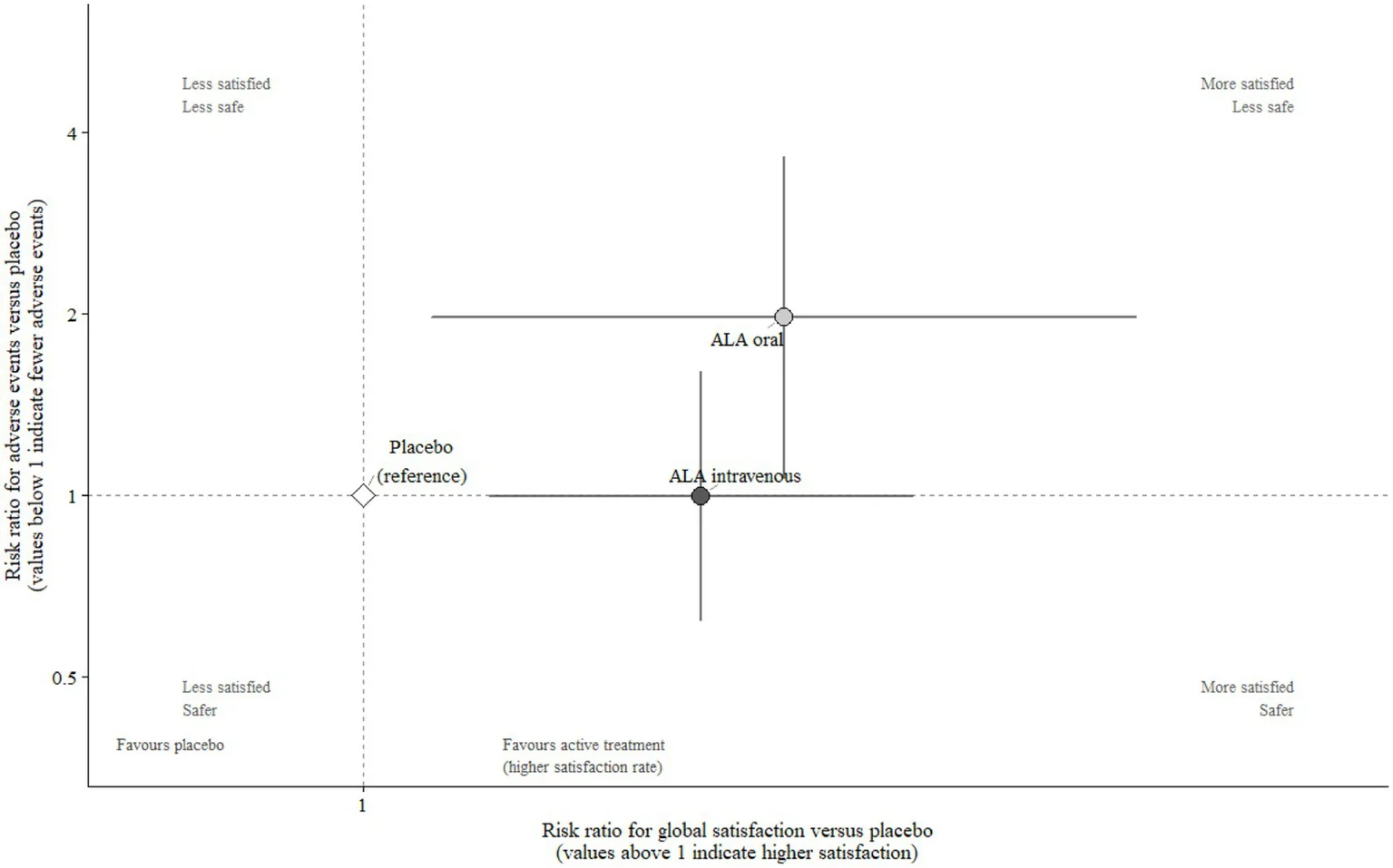
The dual-outcome quadrant plots for GS.

Conversely, although oral ALA therapy possessed a marginally superior point estimate for satisfaction, its position on the Y-axis hovered within the upper quadrant. This spatial distribution elucidates the distinct rationales driving patient benefit: certain patients favor the minimal side-effect profile associated with intravenous therapy, whereas others prioritize the convenience of oral administration. Ultimately, both active approaches achieved comparable utility, substantially elevating overall patient satisfaction. The relevant Dual-outcome quadrant plots is detailed in Figure 21.

### 3.5 Publication bias and evidence quality

To evaluate publication bias and small-study effects, comparison-adjusted funnel plots were constructed. In NMA, the asymmetry of this plot is assessed based on a specific, meaningful sequence of treatments. We established the reference axis in the order of “Placebo → Oral → Sequential → Intravenous.” This sequence was strictly defined based on an escalation of treatment intensity, clinical invasiveness, and expected acute efficacy, rather than simple dose increments. The underlying rationale is that small-study effects typically tend to exaggerate the effect sizes of more invasive or intensively anticipated interventions compared to standard or inactive ones. Furthermore, a quantitative assessment was performed using the Egger’s regression test analogue for NMA.

#### 3.5.1 TSS

We generated a comparison-adjusted funnel plot to evaluate the presence of small-study effects and potential publication bias within the NMA of the TSS. To ensure the comparability of effect sizes across heterogeneous studies, the funnel plot was calibrated symmetrically around the vertical zero line in accordance with the predefined intervention hierarchy: Placebo → ALA oral → ALA sequential → ALA intravenous.

The scatter points representing individual pairwise comparisons were predominantly concentrated in the upper and middle regions of the plot—an area corresponding to smaller standard errors and larger sample sizes—and were distributed symmetrically around the central vertical axis. This spatial distribution suggests the absence of severe small-study effects or significant publication bias within this network model.

**Figure 22:**
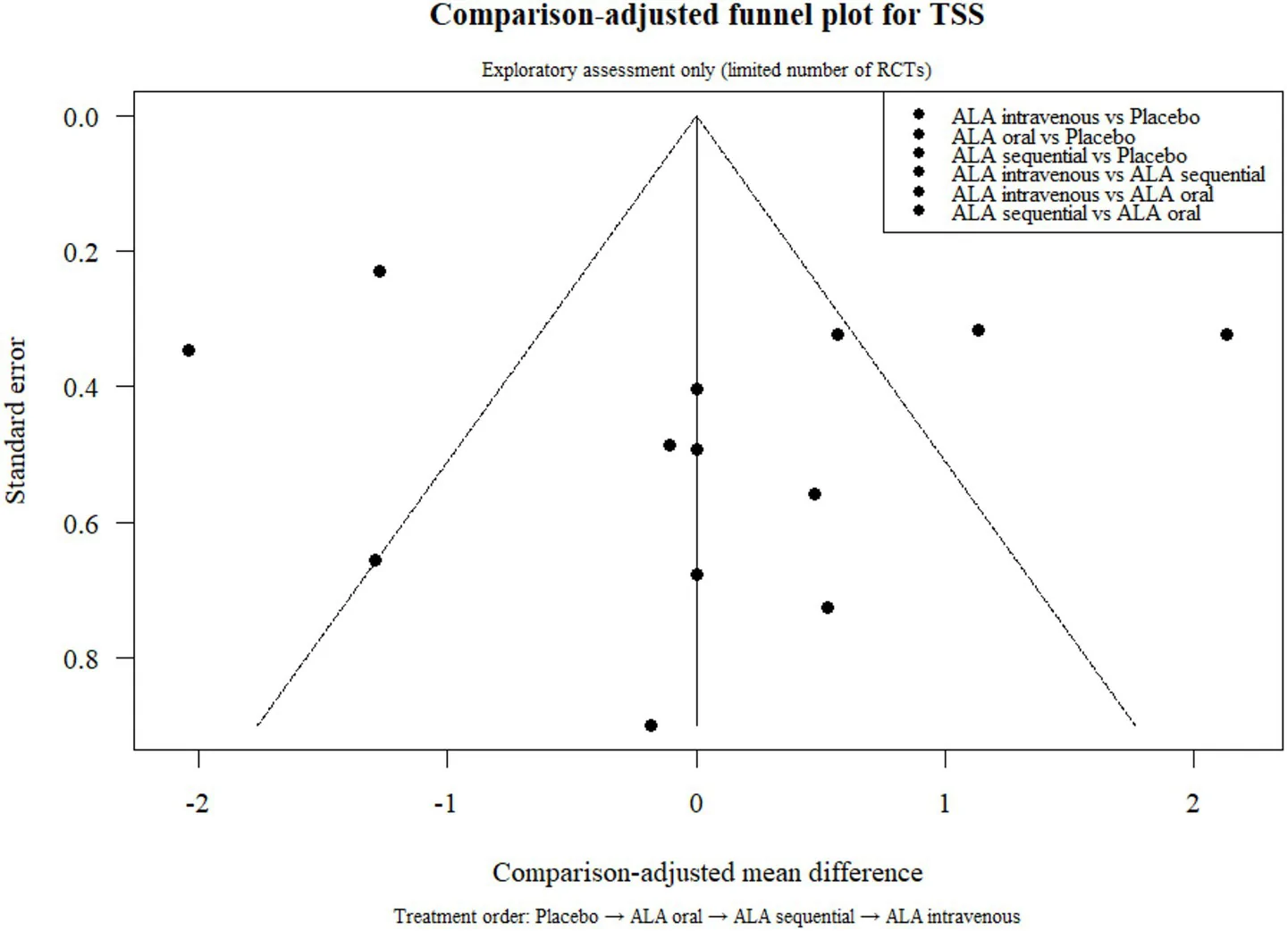
Publication bias and evidence quality for TSS.

However, while the majority of data points fell within the 95% pseudo-confidence intervals delineated by the diagonal lines, a few outliers deviated beyond the boundaries of the inverted funnel, particularly around the mean difference (MD coordinates of −2.0 and +2.0). This departure indicates a certain degree of heterogeneity in effect size estimations among the included studies, which may be attributable to clinical baseline discrepancies, such as ALA dosages ranging from 600 to 1,800 mg/day and follow-up durations spanning from several weeks to multiple years. The relevant figure of publication Bias and Evidence Quality is detailed in Figure 22.

#### 3.5.2 NIS

In the funnel plot for the NIS, the individual scatter points were distributed symmetrically on both sides of the central vertical reference line, with all effect size points residing within the 95% pseudo-confidence interval boundaries. The scatter points representing the various comparisons were predominantly clustered in the middle-to-lower regions of the funnel plot, with standard errors concentrated between 0.6 and 1.0.

**Figure 23:**
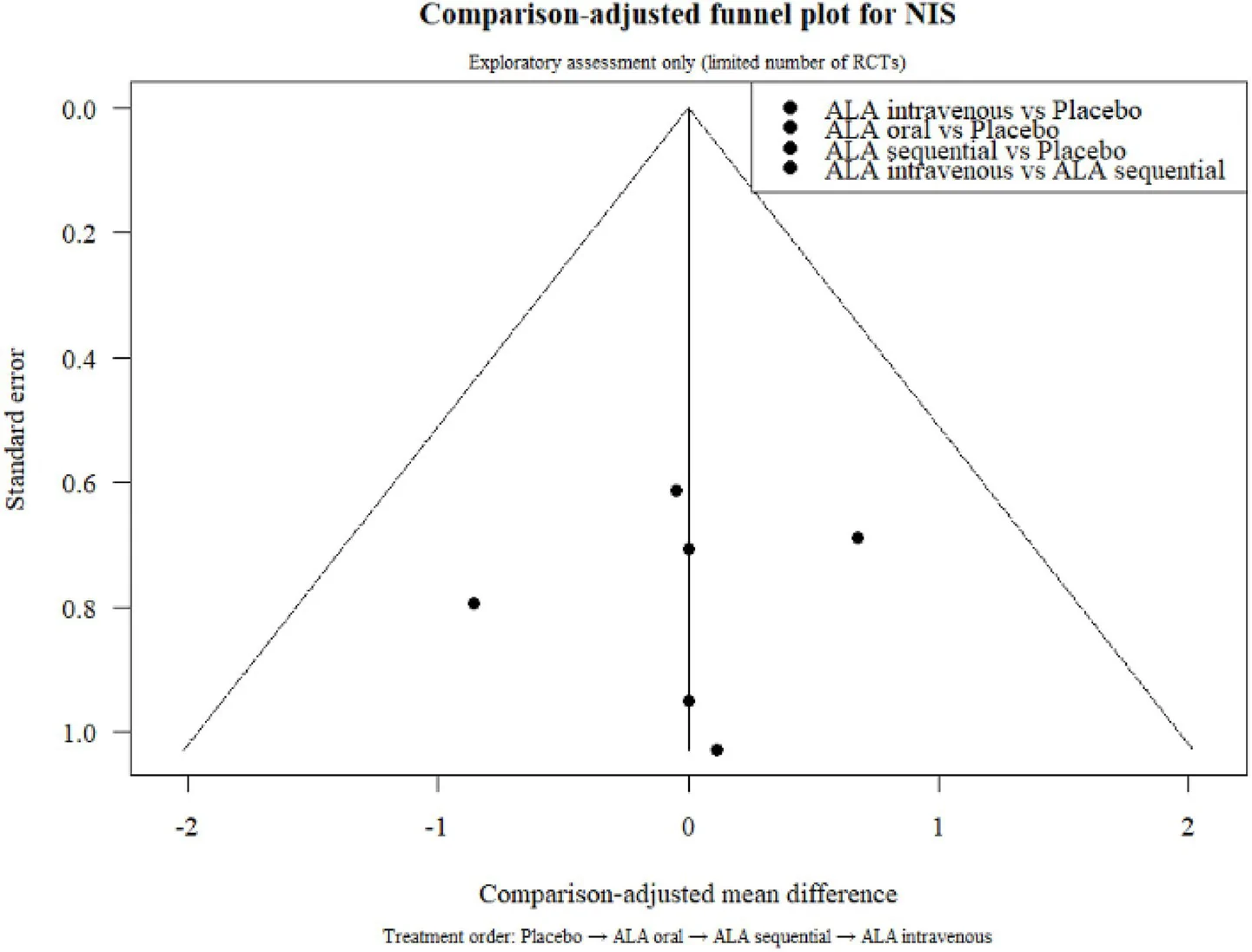
Publication bias and evidence quality for NIS.

This distribution reflects the fact that, compared to subjective symptom scales that merely require questionnaire completion, NIS measurements are more labor-intensive and require specialized neurological expertise, which inherently constrains the sample sizes of individual trials. Given the limited number of RCTs reporting this outcome, this assessment is strictly defined as an exploratory evaluation in adherence to the Cochrane guidelines. The relevant figure of publication Bias and Evidence Quality is detailed in Figure 23.

Visual inspection of the comparison-adjusted funnel plots for TSS and NIS did not reveal obvious asymmetry. To robustly quantify this, we performed the Egger’s regression test. For the TSS outcome, to meet the *Cochrane Handbook* recommendation that at least 10 studies are required for adequate statistical power, the test was conducted on a broader pre-exclusion dataset comprising 13 comparisons. This yielded a *p*-value of 0.721 (*t* = 0.37, df = 11). Similarly, the quantitative Egger’s test for the NIS outcome yielded a *p*-value of 0.763 (*t* = −0.32, df = 4). Both tests consistently provided quantitative evidence supporting the absence of significant publication bias or small-study effects in our network.

#### 3.5.3 NISLL

Because the network comparative framework for the NIS-LL comprises only three RCTs, the number of independent comparative scatter points in the plot is inherently sparse. These points are primarily located in the middle-to-lower sections of the funnel plot, indicating that the individual studies possess relatively small sample sizes and correspondingly larger standard errors.

**Figure 24:**
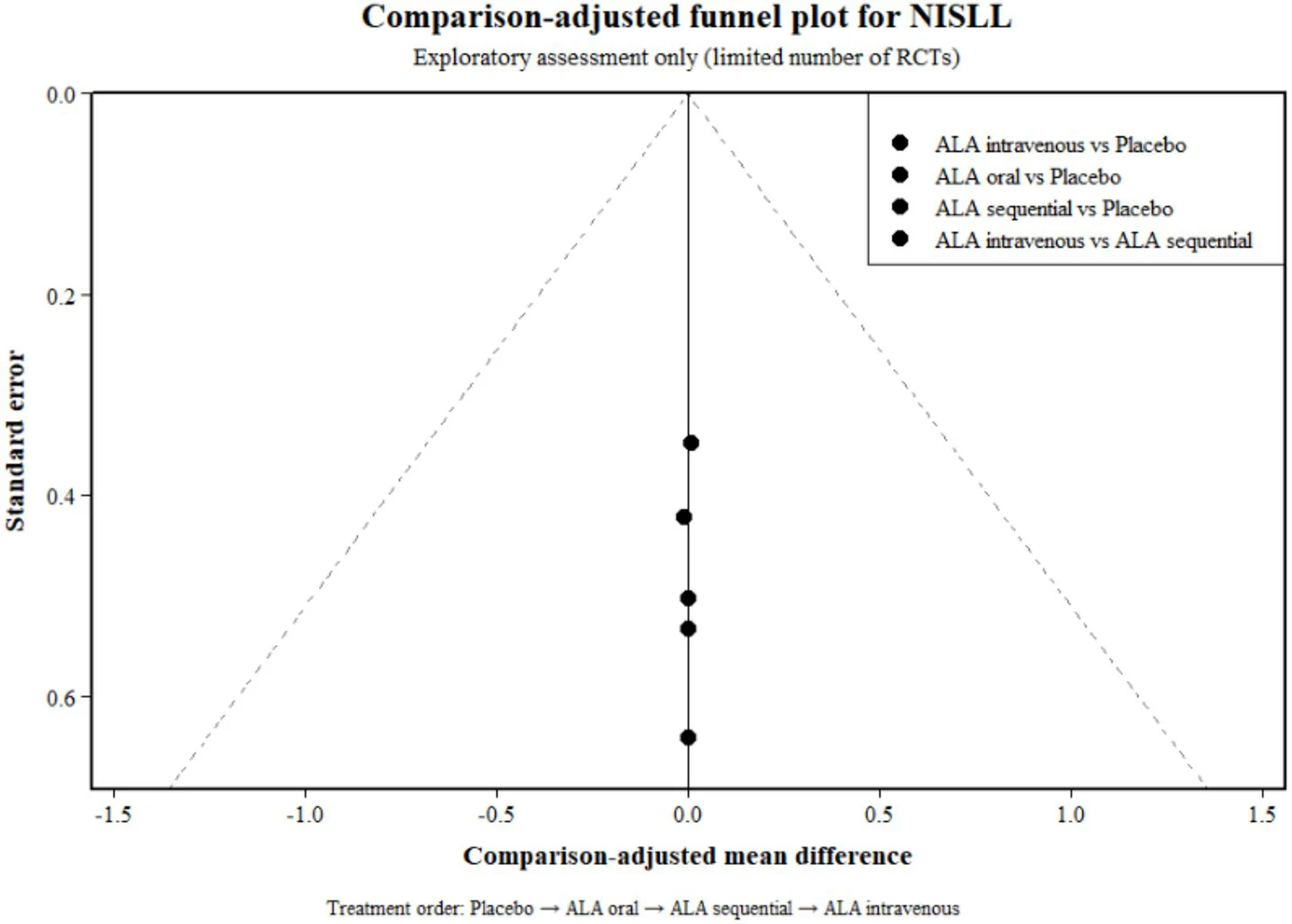
Publication bias and evidence quality for NISLL.

Although the scatter points generally did not deviate significantly from the 95% pseudo-confidence intervals, they lack sufficient statistical power to definitively confirm or refute the presence of publication bias or small-study effects through visual symmetry assessment or quantitative methods such as Egger’s test. Consequently, the comparison-adjusted funnel plot for the NIS-LL serves as a purely exploratory visual presentation. This sparse distribution underscores the relative paucity of high-quality, head-to-head clinical evidence targeting length-dependent distal nerve impairment. The relevant figure of publication Bias and Evidence Quality is detailed in Figure 24.

#### 3.5.4 GS

Regarding the GS outcome, the network evidence structure lacks closed loops and incorporates a limited number of individual RCTs. In accordance with the Cochrane Handbook and the PRISMA-NMA reporting guidelines, when an evidence network is highly sparse and lacks sufficient connectivity, neither visual symmetry assessments nor quantitative statistical tests yield adequate statistical power. Therefore, systematic evaluations for small-study effects or publication bias were omitted for the GS outcome.

Finally, we regenerated the comparison-adjusted funnel plots for the sensitivity analyses. While the exclusion of studies with a high risk of bias naturally reduced the number of scatter points—most notably in the NIS-LL network, which collapsed to a mere two nodes—the remaining high-quality studies for TSS and NIS maintained a generally symmetrical distribution. This confirms that the dramatic shifts observed in our sensitivity analyses were driven by the elimination of methodological bias, rather than the introduction of small-study effects.

### 3.6 CINeMA

To further clarify the robustness of the evidence underlying the SUCRA rankings, this study employed the CINeMA (Confidence in Network Meta-Analysis) framework to conduct a comprehensive certainty of evidence assessment for the four core outcomes (GS, NIS-LL, TSS, and NIS).

The CINeMA evaluations for TSS and NIS revealed the pervasive limitations of the historical literature in this field. The assessment matrix displayed numerous ‘Major concerns,’ primarily concentrated in the domains of ‘Imprecision’ and ‘Within-study bias.’ Particularly for comparisons involving sequential therapy, the certainty ratings were mostly ‘Very low’ due to severe within-study bias. Meanwhile, the TSS network suffered from widespread imprecision driven by limited sample sizes and high heterogeneity, leading to downgraded ratings. For the NIS outcome, only the oral ALA versus placebo comparison maintained a ‘Moderate’ certainty rating. These objective ratings effectively explain the wide 95% confidence intervals observed for certain interventions.

The CINeMA results for NIS-LL exhibited a pronounced polarization, perfectly corroborating the findings of our sensitivity analysis. All comparisons involving intravenous and sequential therapies displayed ‘Major concerns’ in the ‘Within-study bias’ domain, reducing their overall certainty of evidence to ‘Low’ or ‘Very low.’ In stark contrast, the evidence quality for oral ALA versus placebo performed exceptionally well across all six evaluation domains with ‘No concerns,’ achieving an overall rating of ‘High.’ This establishes oral ALA as the sole intervention possessing a high-quality evidence base for ameliorating distal lower-extremity signs.

Although the GS network lacked closed loops, owing to the robustness of the direct comparative evidence, the certainty ratings for both intravenous ALA versus placebo and oral ALA versus placebo achieved ‘High,’ with ‘No concerns’ across all evaluation domains (e.g., bias, imprecision, and heterogeneity). While the intravenous versus oral comparison was based solely on indirect evidence—resulting in ‘Some concerns’ regarding imprecision—its overall rating was nevertheless maintained at ‘High.’ This confirms the core value of ALA therapies in enhancing global patient satisfaction.

## 4 Discussion

### 4.1 Summary of core findings

This study addresses a critical clinical knowledge gap regarding the comparative efficacy and safety of different ALA administration routes. Utilizing a NMA framework, we provide the first quantitative synthesis of the overall performance of oral, IV, and sequential ALA therapies for DPN. Based on our SUCRA rankings and pooled network effect sizes, sequential ALA therapy—characterized by initial short-term IV intensive treatment followed by oral maintenance—emerged as the optimal regimen for ameliorating both subjective symptoms and objective signs, including the TSS, NIS, and NIS-LL.

Concurrently, standalone oral ALA therapy serves as a suboptimal yet conclusively supported foundational approach, backed by robust direct and indirect evidence. In contrast, standalone short-term IV administration exhibited a placebo-like lack of efficacy when addressing the most refractory distal lower-extremity signs (NIS-LL). These rankings not only align with landmark clinical trials but are also supported by distinct pharmacokinetic and biological mechanisms.

However, when the analysis was strictly restricted to high-quality RCTs free from a high risk of bias, a novel paradigm emerged: intravenous ALA therapy stood out as the absolute superior intervention for the rapid amelioration of subjective symptoms (TSS) and overall objective signs (NIS), achieving a ‘dual-optimal’ efficacy-safety profile. Conversely, as the sole regimen supported by high-quality evidence, oral ALA therapy established its unshakeable status as the foundational cornerstone for reversing refractory distal lower-extremity signs (NIS-LL) and facilitating long-term structural repair.

### 4.2 Mechanistic explanations and contextualization with literature

The differentiated efficacy rankings and the spatial coordinate shifts observed across the various administration routes reflect the complex neuropathological progression of DPN and the unique pharmacokinetic profiles of ALA.

#### 4.2.1 The “Short-Term Intensive” nature of intravenous administration and rapid restoration of neural blood flow

From a pharmacokinetic perspective, IV administration bypasses gastrointestinal absorption limits and hepatic first-pass metabolism, enabling ALA to achieve substantially higher systemic exposure within a short timeframe. Compared to oral administration, this route more rapidly elevates effective drug concentrations in the plasma and tissues, thereby exerting potent short-term antioxidant and vasoprotective effects (21). The early symptoms of DPN are not entirely driven by irreversible axonal loss; a significant proportion stems from reversible dysfunction, encompassing endoneurial ischemia, oxidative stress, mitochondrial energy metabolic failure, reduced Na^+^/K^+^-ATPase activity, and compromised neural blood flow. High concentrations of the active drug rapidly cross cell membranes, highly efficiently neutralizing free ROS in blood and tissues and upregulating the expression of endothelial nitric oxide synthase. This rapid improvement in vascular endothelial function directly dilates the previously ischemic neural microvessels, thereby swiftly alleviating subjective symptoms induced by neural ischemia, such as burning pain and numbness, as captured by the TSS (22). The ALADIN study corroborated this, demonstrating that 3 weeks of IV ALA therapy at 600 mg/day significantly reduced DPN symptom scores, indicating a distinct advantage of short-term IV treatment for symptom amelioration (14).

However, DPN is fundamentally a length-dependent disease characterized by axonal degeneration and demyelination (23). Although short-term IV therapy can rapidly mitigate oxidative stress and restore neural blood flow, its duration may be insufficient to facilitate axonal regeneration, myelin remodeling, and the functional recovery of distal nerve conduction. Objective neurological signs, such as the NIS and NIS-LL, reflect deeper structural and functional neurological deficits (24), which are far more recalcitrant to short-term modification than the TSS. This elucidates why standalone short-term IV administration presented a placebo-like failure when confronting the most intractable distal lower-extremity objective signs (NIS-LL). Once the infusion ceases, the distal nerve fibers—deprived of their supportive antioxidant microenvironment—relapse into metabolic derangement (25). Systematic reviews have noted that while ALA provides more consistent evidence for improving pain, burning, and paresthesia, its impact on nerve conduction velocity and objective neurological deficits exhibits heterogeneity. This suggests that its efficacy may be constrained by disease duration, treatment length, and the underlying severity of nerve injury (26).

Our sensitivity analysis perfectly corroborates this pharmacokinetic reality. Studies with a high risk of bias—frequently utilizing open-label designs—likely inflate the perceived efficacy of oral formulations in ameliorating subjective symptoms via psychological expectations and placebo effects. Once this methodological bias is eliminated, intravenous therapy decisively establishes its dominance in both the TSS and NIS outcomes. Intravenous administration immediately achieves supraphysiologic plasma concentrations, facilitating a rapid metabolic reset. This process rapidly eradicates oxidative stress and restores endoneurial microvascular perfusion—mechanisms that acutely alleviate burning pain and sensory deficits without incurring gastrointestinal adverse events.

#### 4.2.2 The “Induction-Maintenance” synergistic remodeling mechanism of sequential therapy

This study demonstrates that sequential therapy excels in improving objective neurological signs, a finding best explained by the “induction-maintenance” model. The initial IV therapy primarily fulfills an “induction phase” role, rapidly reducing the oxidative stress burden, restoring endothelial function and neural blood flow, and partially correcting neural energy metabolic dysfunction in the short term. Subsequently, oral ALA fulfills the “maintenance phase” role, prolonging the favorable neural microenvironment established by the initial IV therapy through sustained and stable antioxidant coverage.

This continuous treatment paradigm likely confers multi-layered synergistic effects. First, the sustained reduction of ROS can mitigate lipid peroxidation, protein glycation, and mitochondrial damage (27, 28). Second, the long-term enhancement of NO bioavailability and microcirculatory perfusion assists in maintaining endoneurial oxygen supply (29). Third, a stable metabolic environment may foster the functional recovery of Na^+^/K^+^-ATPase, improving axolemmal excitability and nerve conduction (30). Fourth, prolonged antioxidant support likely facilitates Schwann cell functional recovery, myelin maintenance, improved axoplasmic transport, and distal axonal regeneration (31, 32).

In other words, IV therapy serves as a “rapid metabolic reset,” whereas oral maintenance functions as “chronic pathological microenvironment management.” Neural repair in DPN operates on a timescale of months rather than days or weeks. Consequently, sequential therapy uniquely covers both critical therapeutic windows in DPN: the short-term window for symptom relief and the long-term window for structural repair. This elucidates why sequential therapy outperforms standalone short-term IV or oral therapies.

The sensitivity analysis resulted in the complete collapse of the sequential therapy node, exposing a severe paucity of high-quality, double-blind RCTs supporting this regimen. However, from a biological standpoint, the ‘induction-maintenance’ model of sequential therapy remains highly plausible: intravenous therapy serves as a ‘rapid metabolic reset,’ whereas oral maintenance functions as ‘chronic pathological microenvironment management.’ Ultimately, the disappearance of sequential therapy from the unbiased network highlights a profound gap in evidence-based medicine, rather than a failure of its biological rationale.

#### 4.2.3 The oral cornerstone and the “Gastrointestinal-Satisfaction” trade-off in the dual-outcome plot

Regarding the strategy of relying on long-term oral ALA as foundational therapy, it achieved significant improvements across all continuous variable networks (TSS, NIS, NIS-LL), affirming the indispensability of long-term antioxidant coverage in reversing distal neuropathy (NIS-LL). Although standalone oral ALA struggles to reach the early peak concentrations of IV administration, its primary advantage lies in delivering long-term, sustained antioxidant coverage. For a chronic, progressive condition like DPN, prolonged, low-intensity but steady suppression of oxidative stress is likely more advantageous for delaying distal nerve damage progression than complete treatment cessation following short-term, high-intensity therapy (33).

Nevertheless, the unique “efficacy-safety” dual-outcome quadrant plot in this study exposes the genuine clinical dilemma of the oral route. Classic large-scale trials, such as SYDNEY 2, have demonstrated that oral ALA (especially in the 1,200 mg or 1,800 mg/day high-dose exploratory arms) provokes dose-dependent gastrointestinal adverse events (18). Owing to the local gastric mucosal irritant properties of oral formulations, the incidence of nausea and vomiting significantly increases among patients (34). This explains why the adverse event coordinate for standalone oral administration is suspended high in the upper quadrant of the dual-outcome plot. Conversely, IV administration perfectly bypasses gastrointestinal irritation by entering the bloodstream directly, achieving “zero additional gastrointestinal risk,” thereby attaining an equivalently high level of psychological satisfaction for patients.

In summary, the various ALA dosing regimens are not simply delineated by variations in absolute efficacy; rather, they reflect differing degrees of alignment with distinct pathological stages of DPN. IV administration is optimally suited for rapidly ameliorating functional symptoms triggered by oxidative stress, endothelial dysfunction, and neural hypoperfusion. Oral therapy is more appropriate for maintaining a prolonged antioxidant and metabolically stable environment. Meanwhile, sequential therapy likely drives simultaneous symptom relief and objective neurological improvement through a dual-phase mechanism of “rapid induction + long-term maintenance.”

The sensitivity analysis resulted in the complete collapse of the sequential therapy node, exposing a severe paucity of high-quality, double-blind RCTs supporting this regimen. However, from a biological standpoint, the ‘induction-maintenance’ model of sequential therapy remains highly plausible: intravenous therapy serves as a ‘rapid metabolic reset,’ whereas oral maintenance functions as ‘chronic pathological microenvironment management.’ Ultimately, the disappearance of sequential therapy from the unbiased network highlights a profound gap in evidence-based medicine, rather than a failure of its biological rationale.

### 4.3 Clinical implications

Based on the multidimensional evidence rankings and the dual-outcome quadrant trade-offs provided by this NMA, we propose a stratified, evidence-based intervention strategy to guide clinical practice for DPN:

1. *Severe neurological deficits*: For patients with severe disease or markedly impaired objective neurological signs (high baseline NIS scores), sequential ALA therapy is the primary recommendation. Our network evaluation confirms that this regimen holds a decisive advantage in reversing the NIS (MD = −2.14, SUCRA: 85.5%), maximizing the substantive restoration of neuroanatomical structures. The initial intensive intravenous phase of sequential therapy delivers maximal and rapid neurovascular rescue while bypassing gastrointestinal irritation, thereby achieving a ‘dual-optimal’ safety profile.
2. *Refractory distal involvement*: For patients presenting with length-dependent distal nerve damage (high NIS-LL) requiring long-term maintenance, standard-dose (600 mg/day) oral ALA therapy should be established as the foundational regimen. Confronting the most refractory distal lower-extremity damage, standalone short-term IV administration proved ineffective (MD = 0.05), whereas long-term oral therapy was the sole route to sustain a robust benefit for the NIS-LL (MD = −0.78) while enhancing global patient satisfaction (RR = 1.28).
3. *Personalized patient care*: Dosing regimens must be tailored according to individual tolerability. For patients who experience severe gastrointestinal intolerance from oral formulations, clinicians can leverage the pharmacokinetic advantage of IV therapy, which carries minimal additional gastrointestinal risk. This route can be utilized as a short-term bridging regimen to alleviate acute subjective symptoms (TSS), thereby safeguarding treatment adherence and patient well-being.

### 4.4 Study limitations and future perspectives

First, the evidence network is topologically sparse and suffers from imprecision. Driven by stringent quality control, our network ultimately incorporated only nine RCTs, resulting in a paucity of large-sample, head-to-head comparisons. Consequently, for outcomes like NIS-LL and GS, the networks exhibited V-shaped topologies or lacked closed loops entirely. Specifically, supported by merely three RCTs, sequential therapy for NIS-LL yielded a widened confidence interval crossing the line of no effect (MD = −1.02). This imprecision reflects insufficient statistical power stemming from a lack of direct evidence, rather than definitive clinical inefficacy.

Second, the evidence base supporting sequential therapy is methodologically fragile. Up to 44.4% of the included original studies carried a high risk of bias according to the Cochrane RoB 2.0. Notably, when assessing highly subjective scales (e.g., TSS and GS), early trials often lacked rigorous double-blind designs or proper intention-to-treat (ITT) analyses. Our rigorous CINeMA evaluation consequently downgraded the overall certainty of evidence for sequential therapy to ‘Low’ or ‘Very low’ due to within-study bias and imprecision. Strikingly, excluding high-risk studies in our sensitivity analysis precipitated the complete collapse of the sequential therapy node, reducing the NIS-LL network to a solitary comparison between oral ALA and placebo. This strongly implies a critical reality: the clinically lauded superiority of sequential therapy is largely an artifact driven by the biases of early, underpowered studies, highlighting a profound vulnerability in contemporary DPN treatment paradigms.

Third, objective constraints limited the quantitative assessment of publication bias. According to the Cochrane Handbook, quantitative tests for funnel plot asymmetry require at least 10 studies to ensure adequate statistical power. With only nine RCTs included, robust quantitative assessment was constrained. Although we conducted exploratory Egger’s tests on a broader pre-exclusion dataset for TSS (k = 13, *p* = 0.721) and the core NIS network (k = 6, *p* = 0.763)—both indicating no overt publication bias—the potential presence of small-study effects cannot be definitively ruled out. These results must therefore be interpreted with caution.

Finally, there are limitations regarding outcome measure selection in the original trials. While scales like the TSS and NIS remain staples in classic RCTs, they are inherently subjective. Recent advancements underscore a critical transition toward high-resolution, objective biomarkers. To complete and strengthen the current evidence network, future multicenter, head-to-head, double-blind RCTs (particularly comparing oral, intravenous, and sequential ALA therapies) must integrate multimodal strategies—including serum neurofilament light chain (NfL), corneal confocal microscopy (CCM), and quantitative sensory testing (QST)—to facilitate the early and precise detection of nerve repair.

## 5 Conclusion

ALA is a safe and effective therapeutic agent for DPN. By utilizing CINeMA evaluations and sensitivity analyses to eliminate historical methodological biases, this network meta-analysis redefines the clinical application of ALA into a stage-specific paradigm. Intravenous ALA provides unmatched, “dual-optimal” efficacy for acute symptom relief and functional rescue while bypassing gastrointestinal risks. Conversely, long-term oral ALA stands as the sole, rigorously verified foundational therapy for reversing refractory distal structural neuropathy.

Crucially, these phase-specific advantages profoundly validate the “induction-maintenance” rationale of sequential therapy. The proven superiority of IV induction, seamlessly complemented by enduring oral maintenance, robustly justifies this clinical strategy. While unbiased direct evidence evaluating the integrated sequential regimen remains sparse—necessitating future rigorous, double-blinded RCTs—the sequential framework emerges as a highly rational, scientifically grounded paradigm for DPN management.

## Data Availability

The original contributions presented in the study are included in the article/Supplementary material, further inquiries can be directed to the corresponding author.
