# Supplementary material for: Oral, intravenous, or sequential alpha-lipoic acid for diabetic peripheral neuropathy? A Bayesian network meta-analysis of randomized controlled trials
Source: Front Neurol. 2026 Aug 11;17:1904967. doi: 10.3389/fneur.2026.1904967 (PMC13503561; doi:10.3389/fneur.2026.1904967)
Supplement: Supplementary file 1 [file Table_2.DOCX]

Search Strategy

1. Embase

('diabetic neuropathy'/exp OR 'diabetic neuropathy':ti,ab,kw OR 'diabetic neuropathies':ti,ab,kw OR 'diabetic peripheral neuropathy':ti,ab,kw OR 'diabetic peripheral neuropathies':ti,ab,kw OR 'diabetic polyneuropathy':ti,ab,kw OR 'diabetic polyneuropathies':ti,ab,kw OR 'dpn':ti,ab,kw OR 'dspn':ti,ab,kw) AND ('thioctic acid'/exp OR 'thioctic acid':ti,ab,kw OR 'alpha lipoic acid':ti,ab,kw OR 'lipoic acid':ti,ab,kw OR 'a-lipoic acid':ti,ab,kw OR 'alpha lipoate':ti,ab,kw OR 'lipoate':ti,ab,kw OR 'ala':ti,ab,kw) AND ('randomized controlled trial'/exp OR 'controlled clinical trial'/exp OR 'random allocation'/exp OR 'double blind procedure'/exp OR 'single blind procedure'/exp OR 'crossover procedure'/exp OR 'placebo'/exp OR 'random*':ti,ab,kw OR 'placebo*':ti,ab,kw OR 'trial':ti,ab,kw OR 'groups':ti,ab,kw OR 'double blind':ti,ab,kw OR 'single blind':ti,ab,kw) NOT (('animal'/exp OR 'animal experiment'/exp OR 'nonhuman'/exp) NOT 'human'/exp)

2. Pubmed

(("Diabetic Neuropathies"[Mesh] OR "diabetic neuropathy"[Title/Abstract] OR "diabetic neuropathies"[Title/Abstract] OR "diabetic peripheral neuropathy"[Title/Abstract] OR "diabetic peripheral neuropathies"[Title/Abstract] OR "diabetic polyneuropathy"[Title/Abstract] OR "diabetic polyneuropathies"[Title/Abstract] OR "DPN"[Title/Abstract] OR "DSPN"[Title/Abstract]) AND ("Thioctic Acid"[Mesh] OR "thioctic acid"[Title/Abstract] OR "alpha-lipoic acid"[Title/Abstract] OR "alpha lipoic acid"[Title/Abstract] OR "lipoic acid"[Title/Abstract] OR "a-lipoic acid"[Title/Abstract] OR "ALA"[Title/Abstract] OR "alpha-lipoate"[Title/Abstract] OR "lipoate"[Title/Abstract]) AND ("randomized controlled trial"[Publication Type] OR "controlled clinical trial"[Publication Type] OR randomized[Title/Abstract] OR placebo[Title/Abstract] OR "drug therapy"[Subheading] OR randomly[Title/Abstract] OR trial[Title/Abstract] OR groups[Title/Abstract])) NOT ("animals"[Mesh] NOT "humans"[Mesh])

3. Cochrane

#1[mh "Diabetic Neuropathies"]

#2("diabetic neuropathy" OR "diabetic neuropathies" OR "diabetic peripheral neuropathy" OR "diabetic peripheral neuropathies" OR "diabetic polyneuropathy" OR "diabetic polyneuropathies" OR DPN OR DSPN):ti,ab,kw

#3#1 OR #2

#4[mh "Thioctic Acid"]

#5("thioctic acid" OR "alpha-lipoic acid" OR "alpha lipoic acid" OR "lipoic acid" OR "a-lipoic acid" OR "alpha-lipoate" OR "lipoate" OR ALA):ti,ab,kw

#6#4 OR #5

#7#3 AND #6

4. Web of science

#1TS=("Diabetic Neuropathies" OR "Diabetic Polyneuropathies" OR "Diabetic Peripheral Neuropathy" OR "DPN")

#2TS=("Thioctic Acid" OR "Alpha-Lipoic Acid" OR "Lipoic Acid" OR "ALA")

#3TS=("randomized controlled trial" OR "controlled clinical trial" OR "randomized" OR "placebo" OR "clinical trials as topic" OR "randomly" OR "trial")

#1 AND #2 AND #3

5. Scopus

( TITLE-ABS-KEY ( "diabetic neuropath*" OR "diabetic peripheral neuropathy" OR "diabetic polyneuropathy" ) ) AND ( TITLE-ABS-KEY ( "thioctic acid" OR "alpha-lipoic acid" OR "lipoic acid" ) ) AND ( TITLE-ABS-KEY ( "randomi*ed" OR "placebo" OR "clinical trial" ) ) AND NOT TITLE-ABS-KEY ( "autonomic" )
