# Supplementary material for: Oral, intravenous, or sequential alpha-lipoic acid for diabetic peripheral neuropathy? A Bayesian network meta-analysis of randomized controlled trials
Source: Front Neurol. 2026 Aug 11;17:1904967. doi: 10.3389/fneur.2026.1904967 (PMC13503561; doi:10.3389/fneur.2026.1904967)
Supplement: Supplementary file 2 [file Data_Sheet_1.ZIP › Appendix.docx]

Appendix

1. Base data

2. TSS

3. NIS

1

4. NISLL

5. GS
